# Synergistic roles of tristetraprolin family members in myeloid cells in the control of inflammation

**DOI:** 10.26508/lsa.202302222

**Published:** 2023-10-30

**Authors:** Brittany L Snyder, Rui Huang, Adam B Burkholder, Danielle R Donahue, Beth W Mahler, Carl D Bortner, Wi S Lai, Perry J Blackshear

**Affiliations:** 1 Signal Transduction Laboratory, National Institute of Environmental Health Sciences, NIH , Research Triangle Park, Durham, NC, USA; 2 Department of Biochemistry, Duke University Medical Center, Durham, NC, USA; 3 Bioinformatics Support Group, National Institute of Environmental Health Sciences, NIH , Research Triangle Park, Durham, NC, USA; 4 NIH Mouse Imaging Facility , National Institute of Neurological Disorders and Stroke, NIH, Bethesda, MD, USA; 5 Experimental Pathology Laboratories, Inc., Research Triangle Park, Durham, NC, USA; 6 Department of Medicine, Duke University Medical Center, Durham, NC, USA

## Abstract

Members of the tristetraprolin (TTP) family of RNA-binding proteins can bind to and promote the decay of specific transcripts containing AU-rich motifs. ZFP36 (TTP) is best known for regulating pro-inflammatory cytokine expression in myeloid cells; however, its mammalian paralogues ZFP36L1 and ZFP36L2 have not been viewed as important in controlling inflammation. We knocked out these genes in myeloid cells in mice, singly and together. Single-gene myeloid-specific knockouts resulted in almost no spontaneous phenotypes. In contrast, mice with myeloid cell deficiency of all three genes developed severe inflammation, with a median survival of 8 wk. Macrophages from these mice expressed many more stabilized transcripts than cells from myeloid-specific TTP knockout mice; many of these encoded pro-inflammatory cytokines and chemokines. The failure of weight gain, arthritis, and early death could be prevented completely by two normal alleles of any of the three paralogues, and even one normal allele of *Zfp36* or *Zfp36l2* was enough to prevent the inflammatory phenotype. Our findings emphasize the importance of all three family members, acting in concert, in myeloid cell function.

## Abstract (precis)

Simultaneous deficiency of three tristetraprolin family members in myeloid cells resulted in severe inflammation in mice, suggesting that these three proteins act together to prevent inflammation.

## Introduction

Posttranscriptional control of gene expression by RNA-binding proteins is vital to tightly regulate the expression of transcripts encoding cytokines, such as tumor necrosis factor (*Tnf*) mRNA (Baou et al, 2011; Gerstberger et al, 2014; Fu & Blackshear, 2017). Defects in or changes in expression of such regulatory proteins can lead to the abnormal accumulation of specific transcripts, such as inflammatory cytokine and chemokine mRNAs, leading to changes in the levels of their encoded proteins (Patial & Blackshear, 2016; Fu & Blackshear, 2017; Uchida et al, 2019; Makita et al, 2021). Increased levels of cytokines, such as TNF, are associated with the chronic inflammation characteristic of many autoimmune diseases and cancers (Kruglov et al, 2008; Moudgil & Choubey, 2011).

Members of the zinc finger protein 36 (ZFP36) or tristetraprolin (TTP) family of RNA-binding proteins bind to specific transcripts, including cytokine mRNAs, and promote mRNA decay (Sanduja et al, 2011; Brooks & Blackshear, 2013; Wells et al, 2017; Lai et al, 2019c; Makita et al, 2021). TTP family members, defined by the specific organization of the RNA-binding tandem zinc finger domain, have been found in most eukaryotes studied to date, although the number of TTP proteins expressed in each species varies (Blackshear & Perera, 2014; Wells et al, 2017; Lai et al, 2019c). Three TTP genes are conserved in mammals (including mice and humans): *Zfp36*, *Zfp36l1*, and *Zfp36l2*, which code for their respective proteins: ZFP36 or TTP, ZFP36L1, and ZFP36L2 (Lai et al, 1990; Taylor et al, 1996; Stoecklin et al, 2002; Stumpo et al, 2004, 2009). All mammalian TTP family proteins contain a highly conserved RNA-binding domain that binds to its preferred AU-rich binding site within the 3′-untranslated regions (3′-UTR) of specific mRNAs (Lai et al, 2019c). However, genetic KO studies of these three genes in mice have revealed very different spontaneous phenotypes (Taylor et al, 1996; Stumpo et al, 2004, 2009).

For example, ZFP36 (TTP) binds to and promotes the decay of many pro-inflammatory cytokine mRNAs, and plays an important role in regulating the expression of the encoded cytokines and chemokines (Carballo et al, 1998; Datta et al, 2008; Baou et al, 2011; Molle et al, 2013; Andrianne et al, 2017; Fu & Blackshear, 2017). *Zfp36*-KO mice develop a severe inflammatory phenotype characterized by myeloid hyperplasia, arthritis, failure of weight gain, and autoimmunity (Taylor et al, 1996). The *Zfp36*-KO phenotype is primarily caused by the altered synthesis and secretion of inflammatory cytokines, such as TNF, in many cells, including myeloid cells, with macrophages being one of the important cellular sources that contribute to the TNF overproduction in *Zfp36*-KO mice (Carballo et al, 1997; Carballo et al, 1998; Carballo & Blackshear, 2001). However, myeloid-specific *Zfp36*-KO (M-TTP KO) mice have a minimal phenotype under normal vivarium conditions at 6–12 wk of age (Kratochvill et al, 2011; Qiu et al, 2012). Nonetheless, M-TTP KO mice are abnormally hypersensitive to LPS and develop a sepsis-like syndrome with greatly elevated serum TNF levels in response to a low-dose LPS challenge (Qiu et al, 2012), under conditions in which control mice are largely unaffected. In addition, BMDM from M-TTP KO mice exhibited abnormal stabilization of TTP target transcripts and increased production of cytokine and chemokine proteins (Qiu et al, 2012).

ZFP36L1 and ZFP36L2 also bind to AU-rich element-containing sequences and promote deadenylation in cell-free deadenylation assays and cell-based transfection assays (Lai et al, 2000, 2003). However, much less is known about the physiological targets of ZFP36L1 and ZFP36L2, most likely because of the early lethality of their phenotypes. Germ line deletion of *Zfp36l1* results in embryonic lethality because of chorioallantoic fusion defects (Stumpo et al, 2004), whereas *Zfp36l2* KO mice die within about 2 wk of birth because of hematopoietic failure (Stumpo et al, 2009). Little is known about the functional importance of *Zpf36l1* and *Zfp36l2* in myeloid cells, although one study showed that myeloid-specific *Zfp36l1* KO mice had no obvious phenotype, and were normally susceptible to models of bacterial pneumonia or lung injury after exposure to Gram-negative bacteria (Hyatt et al, 2014). In general, the potential functions of ZFP36L1 and ZFP36L2 in myeloid cells are poorly understood, particularly in the context of innate immune system activation, in contrast to the well-known role of TTP in these processes.

Recent studies have indicated that deficiency of more than one ZFP36 family member simultaneously in a specific cell-type can have a greater effect than knocking out a single ZFP36 family member in a given cell type (Hodson et al, 2010; Cook et al, 2022). In the present study, we wanted to determine whether *Zfp36*, *Zfp36l1*, and *Zfp36l2* had additive or synergistic functions in myeloid cells, or, potentially, no interactions at all. Accordingly, we generated mice in which all three TTP family member genes (*Zfp36*, *Zfp36l1*, and *Zfp36l2*) were knocked out simultaneously in myeloid cells using *LysM*-Cre (Clausen et al, 1999), referred to herein as M-triple KO mice.

We found that simultaneous deficiency of *Zfp36*, *Zfp36l1*, and *Zfp36l2* in myeloid cells led to the spontaneous development of an early lethal phenotype, with severe arthritis, myeloid hyperplasia, bone resorption, and increased levels of cytokines and chemokines. This phenotype was associated with marked increases in the number of stabilized transcripts found in LPS-stimulated primary macrophages derived from these mice, when compared with cells derived from the M-*Zfp36* KO mice. This phenotype is in stark contrast to the essentially normal phenotypes of single M-*Zfp36* KO, M-*Zfp36l1* KO or M-*Zfp36l2* KO mice under normal vivarium conditions. It is also much more severe, and of earlier onset, than the complete TTP deficiency syndrome (Taylor et al, 1996; Ghosh et al, 2010; Lai et al, 2018). Strikingly, we also found that the external syndrome could be prevented by two normal alleles of any of the three genes, and single normal alleles of at least two genes. These findings suggest that simultaneous deficiencies of *Zfp36*, *Zfp36l1*, and *Zfp36l2* in myeloid cells leads to the synergistic development of an early lethal inflammatory syndrome, at least in part because of excess levels of pro-inflammatory cytokines and chemokines, signifying the importance of all three family members acting in concert in myeloid cells to control the inflammatory response.

## Results

### Analysis of TTP family member mRNA expression in primary BMDM

**Figure 1.**
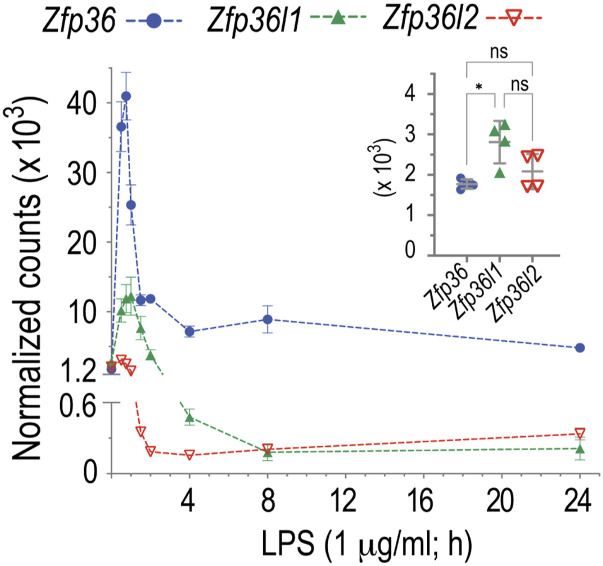
NanoString analysis of BMDM mRNA accumulation. BMDM were incubated with LPS (1 μg/ml) for the times indicated. Total RNA samples prepared from four WT mouse BMDM were used for NanoString analysis. Data are shown as normalized counts (mean ± SD, n = 4) of *Zfp36*, *Zfp36l1*, and *Zfp36l2* mRNA. The inset shows the mRNA counts of samples at time 0 (without LPS stimulation).

To determine normal levels of *Zfp36*, *Zfp36l1*, and *Zfp36l2* mRNAs in WT macrophages before and after LPS treatment, we analyzed BMDM samples from a previous study using NanoString nCounter analysis (see Fig 5A and B in Lai et al [2018]). *Zfp36*, *Zfp36l1*, and *Zfp36l2* mRNAs were present at similar levels in serum-deprived BMDM from WT mice in the absence of LPS, although the average level of *Zfp36l1* mRNA was slightly higher than that of *Zfp36* mRNA (Fig 1, inset). After LPS stimulation, *Zfp36* mRNA levels increased rapidly, peaking at 45 min (23fold increase compared with time 0), and declined thereafter, but remained elevated above basal levels even up to 24 h (Fig 1). *Zfp36l1* mRNA levels briefly increased after LPS treatment, peaking 1 h after LPS (4.4fold increase compared with time 0), followed by a rapid decrease to below baseline levels (Fig 1). *Zfp36l2* mRNA levels also increased slightly, with a peak 1 h after LPS (1.5fold increase compared with time 0), and also rapidly decreased to below baseline values (Fig 1).

Analysis of areas under the curves of these three mRNAs during the 24 h after LPS demonstrated that *Zfp36* mRNA exhibited by far the largest area under the curve, with the *Zfp36l1* and *Zfp36l2* mRNA areas under the curve being 13% and 4% the size of that of *Zfp36* mRNA, respectively.

These data demonstrate that *Zfp36* mRNA was expressed to a much greater extent than the transcripts of the other two TTP family members in primary macrophages after LPS stimulation. This supports the concept that TTP is the major regulator of pro-inflammatory mRNA levels in the context of the acute innate immune response. However, because all three TTP family member mRNAs were expressed at comparable levels in the steady state, each of them could play roles in the control of inflammation in normal physiology or during more chronic inflammation.

### Phenotype of myeloid-specific triple (M-triple) KO mice

To determine whether there were overlapping or synergistic roles of TTP family proteins (ZFP36 or TTP, ZFP36L1, and ZFP36L2) in myeloid cells, we generated myeloid-specific KO mice for each family member individually, and mice in which all three TTP family members were knocked out simultaneously in myeloid cells (*Zfp36*^flox/flox^; *Zfp36l1*^flox/flox^; *Zfp36l2*^flox/flox^; *LysM-Cre*^+/−^), referred to as M-triple KO mice. In this case, we are using the term synergy to indicate that “the effect on survival of combining these mutations is greater than the sum effect of the individual mutations” (Hanson et al, 2019). In the experiments described below, we use the term synergy to indicate that the effects of the M-triple KO mutation were much more severe on mouse survival and other aspects of the phenotype than the sum of the phenotypes from the individual KOs.

**Figure 2.**
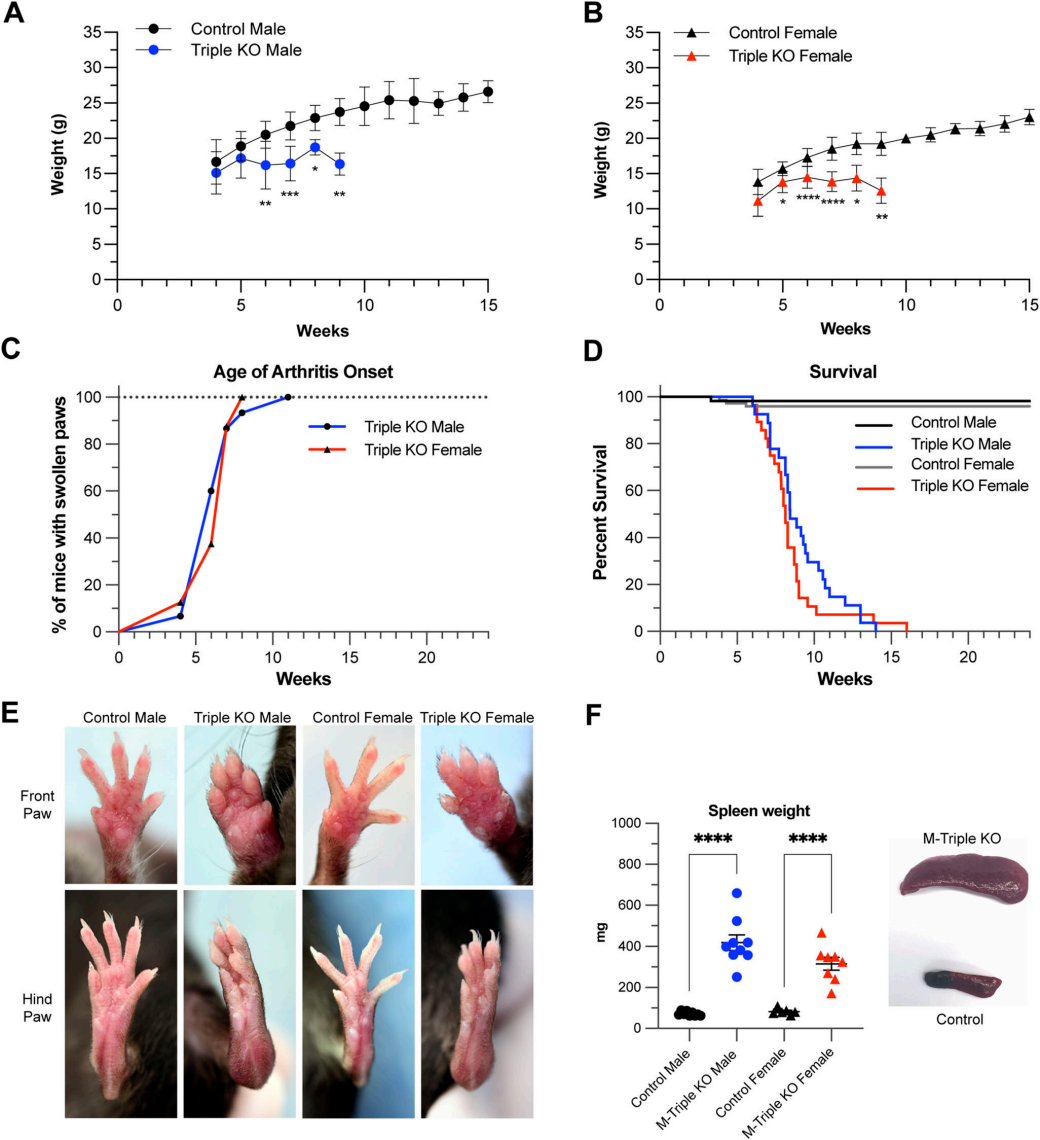
Phenotype of myeloid-specific (M-triple) KO mice. **(A, B)** Growth curves of (A) male control (N = 19) and M-triple KO (N = 14) mice and (B) female control (N = 18) and M-triple KO (N = 20) mice. Mice were weighed weekly, and weights are shown as means ± SD over time (weeks). *P < 0.05; **P < 0.01; ***P < 0.001; ****P < 0.0001, determined by two-way ANOVA with Šídák’s multiple comparison test. **(C)** Mice were monitored weekly for swollen or red paws, and the arthritis prevalence is shown as a percentage of M-triple KO male (N = 15) and M-triple KO female (N = 8) mice with swollen paws at a given age (in weeks). **(D)** Percent survival of control male (N = 55), M-triple KO male (N = 26), control female (N = 74), and M-triple KO female (N = 28) mice at a given age (weeks). **(E)** Photographs of front and hind paws of live male and female control and M-triple KO mice at 10 wk of age. **(F)** Spleen weights shown in mg as mean ± SD, and example photographs of markedly enlarged spleens from M-triple KO mice (bottom) compared to control spleen (top), both from 9-wk-old mice. *P < 0.05; **P < 0.01; ***P < 0.001; ****P < 0.0001, determined by unpaired, two-tailed *t* tests with Welch’s correction.

M-triple KO mice were slightly smaller at birth than control mice, and exhibited slower weight gain, which became obvious at about 5–6 wk of age in both males (Fig 2A) and females (Fig 2B). Failure to gain weight worsened over time in both sexes, and the overall phenotype was generally severe enough to require euthanasia by 9 wk of age (Fig 2A and B). All male and female M-Triple KO mice developed redness and swelling involving all four limbs, with a median age of arthritis onset of 6 wk in both sexes (Fig 2C). The swelling and redness persisted and worsened until death. The most severe swelling was observed in the carpal joints of the front paws and tarsal regions of the hind paws (Fig 2E), but redness and swelling occurred in both the front and hind paws of M-triple KO mice (Fig 2E). In contrast, whole-body TTP KO mice typically have redness and swelling that is more severe in the front limbs and begins much later in life (Taylor et al, 1996). The M-triple KO mice exhibited 100% lethality by 16 wk of age, with a median survival of 8.4 wk for males and 8.1 wk for females (Fig 2D). At necropsy, spleens from both male and female M-triple KO mice were markedly increased in size (Fig 2F).

**Figure S1.**
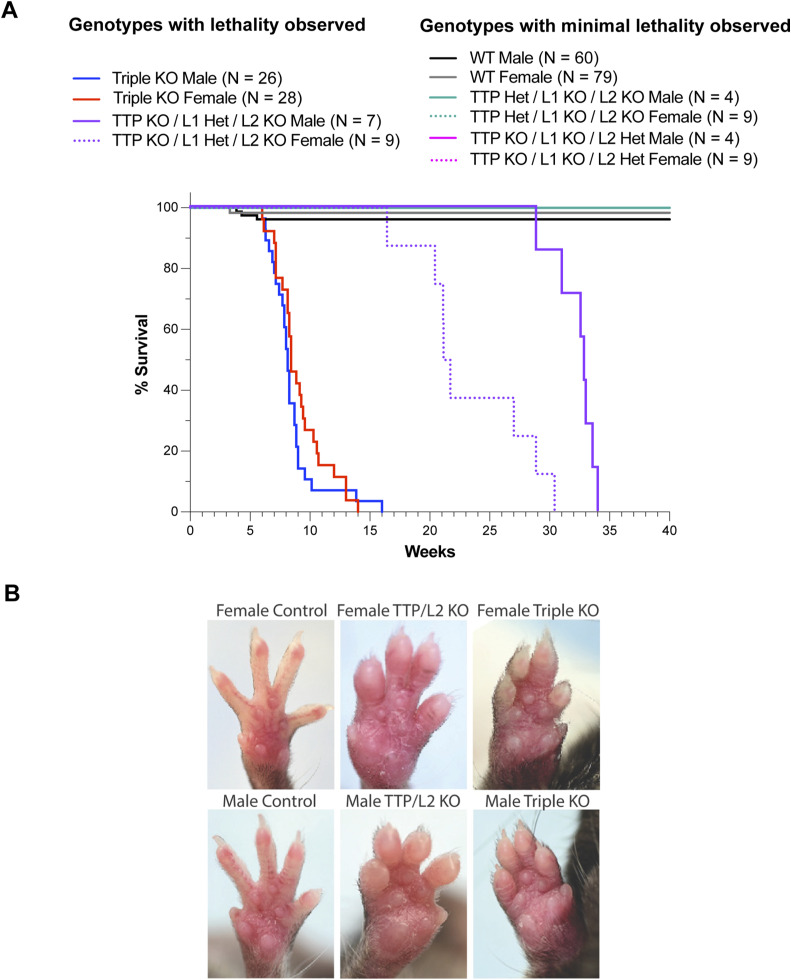
Phenotype of myeloid-specific double KO mice. **(A)** Percent survival of control male (N = 60), M-triple KO male (N = 26), (*Zfp36*^flox/wt^; *Zfp36l1*^flox/flox^; *Zfp36l2*^flox/flox^; *LysM-Cre*^+/−^) male (N = 4), (*Zfp36*^flox/flox^; *Zfp36l1*^flox/wt^; *Zfp36l2*^flox/flox^; *LysM-Cre*^+/−^) male (N = 7), (*Zfp36*^flox/flox^; *Zfp36l1*^flox/flox^; *Zfp36l2*^flox/wt^; *LysM-Cre*^+/−^) (N = 4), control female (N = 79), M-triple KO female (N = 28), (*Zfp36*^flox/wt^; *Zfp36l1*^flox/flox^; *Zfp36l2*^flox/flox^; *LysM-Cre*^+/−^) female (N = 9), (*Zfp36*^flox/flox^; *Zfp36l1*^flox/wt^; *Zfp36l2*^flox/flox^; *LysM-Cre*^+/−^) female (N = 9), and (*Zfp36*^flox/flox^; *Zfp36l1*^flox/flox^; *Zfp36l2*^flox/wt^; *LysM-Cre*^+/−^) female (N = 9) mice at a given age (weeks). Abbreviations for genotypes used in this figure are as follows: control (*Zfp36*^flox/flox^; *Zfp36l1*^flox/flox^; *Zfp36l2*^flox/flox^; *LysM-Cre*^−/−^), M-triple KO (*Zfp36*^flox/flox^; *Zfp36l1*^flox/flox^; *Zfp36l2*^flox/flox^; *LysM-Cre*^+/−^), TTP Het/L1 KO/L2 KO (*Zfp36*^flox/wt^; *Zfp36l1*^flox/flox^; *Zfp36l2*^flox/flox^; *LysM-Cre*^+/−^), TTP KO/L1 Het/L2 KO (*Zfp36*^flox/flox^; *Zfp36l1*^flox/wt^; *Zfp36l2*^flox/flox^; *LysM-Cre*^+/−^), and TTP KO/L1 KO/L2 Het (*Zfp36*^flox/flox^; *Zfp36l1*^flox/flox^; *Zfp36l2*^flox/wt^; *LysM-Cre*^+/−^). **(B)** Photographs of front and hind paws of live male and female controls (24 wk of age), (*Zfp36*^flox/flox^; *Zfp36l1*^flox/wt^; *Zfp36l2*^flox/flox^; *LysM-Cre*^+/−^) (24 wk of age), and M-triple KO (9 wk of age) mice. The abbreviations above refer to the following genotypes: control (*Zfp36*^flox/flox^; *Zfp36l1*^flox/flox^; *Zfp36l2*^flox/flox^; *LysM-Cre*^−/−^), TTP/L2 KO (*Zfp36*^flox/flox^; *Zfp36l1*^flox/WT^; *Zfp36l2*^flox/flox^; *LysM-Cre*^+/−^), and M-triple KO (*Zfp36*^flox/flox^; *Zfp36l1*^flox/flox^; *Zfp36l2*^flox/flox^; *LysM-Cre*^+/−^).

The severe and early lethal phenotype of the M-triple KO mice was in stark contrast to the phenotypes of any of the myeloid-specific single KO mice. Myeloid-specific TTP (M-TTP) KO mice grew to normal size, occasionally developed minor paw swelling after 6 mo of age, and generally survived beyond 8 mo of age (Kratochvill et al, 2011; Qiu et al, 2012) (and data not shown). Myeloid-specific *Zfp36l1* KO (Hyatt et al, 2014) or *Zfp36l2* KO (data not shown) mice grew at normal rates, with no obvious abnormalities at necropsy, and survived beyond 8 mo of age. Mice with myeloid-specific deletion of any two TTP family members together, with two WT alleles of the third, did not develop weight loss or paw swelling or redness and survived beyond 35 wk (data not shown). We also evaluated mice with five of the six family member alleles knocked out, with only one normal allele of one gene remaining. Of these three different combinations, only mice expressing one WT allele of *Zfp36l1*, with all the remaining TTP family alleles deleted in myeloid cells (*Zfp36*^flox/flox^; *Zfp36l1*^flox/WT^; *Zfp36l2*^flox/flox^; *LysM-Cre*^+/−^), developed redness and swelling of the hind paws at about 20 wk of age (Fig S1B), and eventually died prematurely, with a median survival of ∼21 wk for females and ∼32 wk for males (Fig S1A).

### Arthritis and bone loss in M-triple KO mice

**Figure 3.**
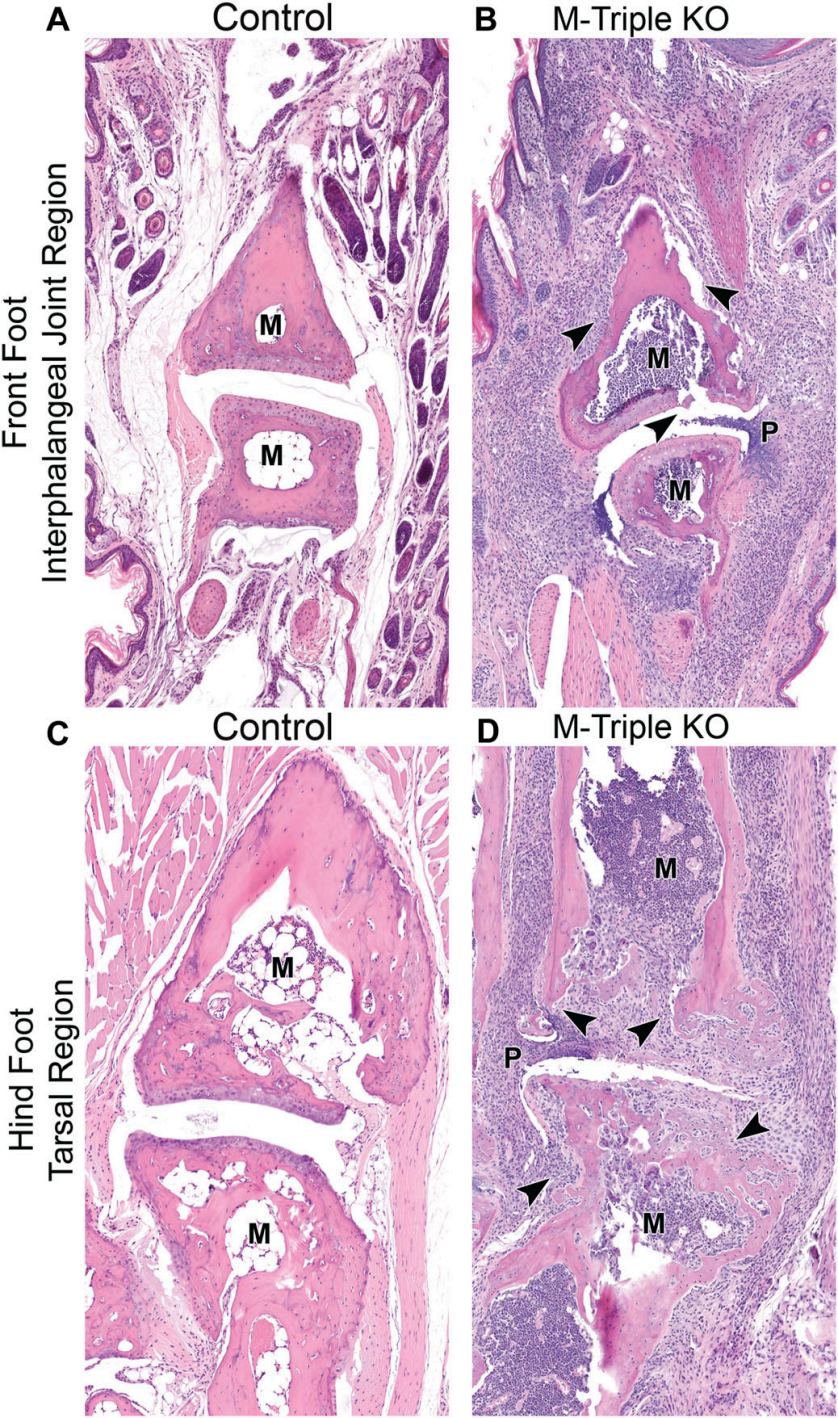
Paw histology. **(A, B)** H&E-stained sections of the interphalangeal joint region of front paws from (A) male control and (B) male M-triple KO mice at 7–9 wk of age (20X magnification). **(C, D)** H&E-stained sections of the metatarsal–tarsal region of the hind foot from (C) male control and (D) male M-triple KO mice at 7–9 wk of age (20X magnification). Arrowheads in the M-triple KO sections indicate neutrophilic infiltration into the bone, cartilage, and soft tissue. Note: marrow cavity (M) is full of myeloid cells in the M-triple KO sections, compared with an essentially empty marrow cavity (M) in the control sections. In the M-triple KO paw sections, the synovium or pannus (P) is protruding into the joint space.

Microscopic analysis of hematoxylin and eosin-stained sections from front and hind limbs from 7- to 10-wk-old male and female M-triple KO mice revealed neutrophilic and/or chronic–active inflammation in the front and hind paws, consisting predominantly of neutrophils and fewer macrophages, admixed with variable amounts of fibrosis, which multifocally expanded and disrupted the soft tissue, bones, and associated joints (Fig 3). Findings were most severe in the interphalangeal joint region in the front limbs (Fig 3B) and the tarsal region in the hind limbs (Fig 3D), when compared with paws from control mice (Fig 3A and C). In contrast, minimal mixed cell inflammation (comprised of neutrophils, and fewer mononuclear cells) was observed focally or multifocally only within the dermis of front paws of M-TTP KO mice; there was no joint and bone involvement.

**Figure 4.**
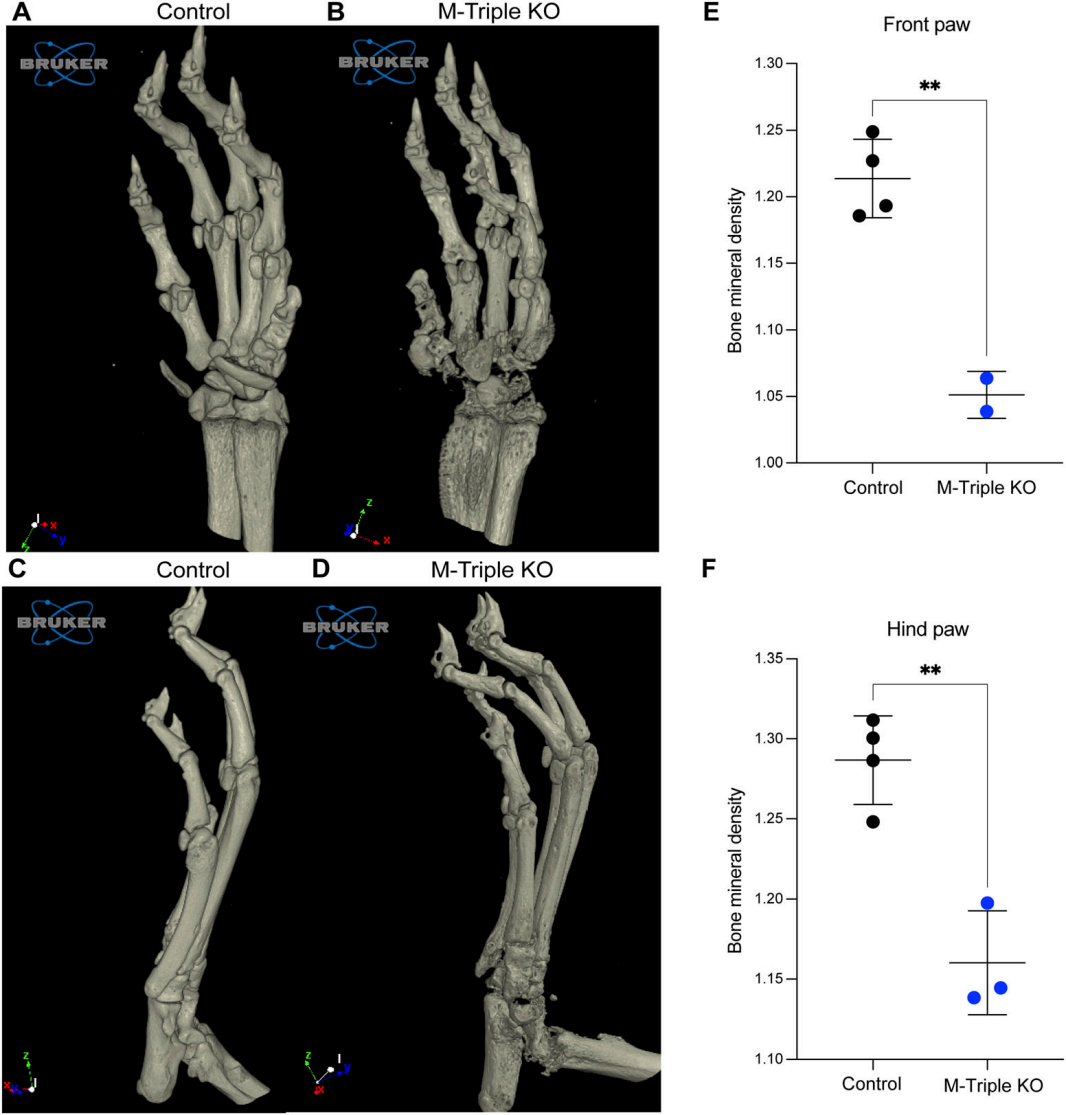
Evaluation of bone architecture and bone mineral density (BMD) of paws from M-triple KO mice. Front and hind paws from 9–10-wk-old male and female control (N = 4) and M-triple KO (N = 3) mice were evaluated for bone erosion by microCT. **(A, B, C, D)** Volume renderings of 6.5 micron isotropic voxel microCT data of front paws from 9-wk-old male (A) control and (B) M-triple KO mice, and hind paws from 9-wk-old male (C) control and (D) M-triple KO mice. **(E, F)** Total BMD of the (E) front and (F) hind paws was calculated from analysis of reconstructed microCT scans using CTAn software and shown as means ± SD. **P < 0.01, determined using unpaired, two-tailed *t* tests with Welch’s correction. More detailed rotational views are included in Video 1 and Video 2.

MicroCT analysis of front and hind limbs from M-triple KO mice demonstrated severe bone erosion and bone loss (Fig 4B and D) when compared with front and hind limbs from control mice (Fig 4A and C). The most significant bone degradation was observed in the carpal and interphalangeal regions of the front paws (Fig 4B and Video 1) and the tarsal and interphalangeal regions of the hind paws (Fig 4D and Video 2). Bone mineral density measurements showed that there were significant decreases in the total average bone mineral density of both the front (Fig 4E) and hind (Fig 4F) paws from M-triple KO mice when compared with WT mice.

Video 1 Download video Movie of 3D volume rendering of MicroCT data of Triple KO front paw. The transfer function of the volume’s opacity has been adjusted to focus on the x-ray attenuation of the bone window, in absence of the soft tissue window, to permit the visualization of the bone structure.

Video 2 Download video Movie of MicroCT data of Triple KO hind paw. Multiple views in order are: (1) 0–42 s: 3D volume rendering with adjusted transfer function of volume’s opacity to the bone window.  Minimal shadowing and light features have been adjusted to accurately present the bone structure detail clearly. (2) 43–46 s: Removal of opacity, shadowing, and lighting adjustments to display native data in 3D format. (3) 47–1 min 11 s: Clipping into volume to allow view of top 2D slice as the clip plane progresses through the 3D volume of the native MicroCT data. (4) 1 min 12 s–1 min 29 s: Smaller subset of clipped slices progressing through the volume to allow focus on the 2D sections of the native MicroCT data. (5) 1 min 30 s–1 min 33 s: Clipping out of the volume to build the 3D volume of the native MicroCT data. (6) 1 min 34 s–1 min 36 s: Clipping out continues as the shadow and lighting features previously used are reapplied to the data. This permits visualization of the data integrity with the applied adjustments. (7) 1 min 37 s–1 min 40 s: Application of the earlier transfer function of the volume’s opacity to the bone window. This permits visualization of the data integrity with the applied adjustment. (8) 1 min 41 s–1 min 45 s: 3D volume rendering with adjusted transfer function of volume’s opacity to the bone window. Minimal shadowing and light features have been adjusted to accurately present the bone structure detail clearly.

These data demonstrate that myeloid-specific deletion of *Zfp36* alone had minimal effects on soft tissue inflammation and did not lead to the development of peripheral arthritis. However, deleting *Zfp36l1* and *Zfp36l2* in addition to *Zfp36* in myeloid cells led to the development of early lethality, accompanied by severe inflammatory arthritis and bone degradation, suggesting that all three TTP family members acted synergistically in the regulation of pro-inflammatory regulators of arthritis and the other aspects of this complex inflammatory syndrome.

### Histopathological evaluation of spleen, liver, lungs, and heart

**Figure S2.**
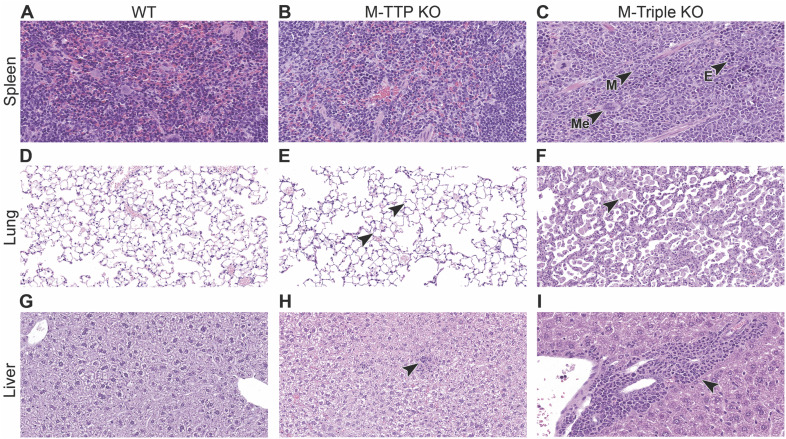
Histopathology of select tissues (lung, liver, spleen). **(A, B, C)** H&E-stained sections of spleens from (A) male control, (B) male M-TTP KO, and (C) male M-triple KO mice at 9 wk of age (40X magnification). Red pulp was expanded by myeloid (M) and fewer erythroid (E) and megakaryocytic (Me) precursors in the M-triple KO spleens. **(D, E, F)** H&E-stained sections of lungs from (D) male control, (E) male M-TTP KO, and (F) male M-triple KO mice at 9 wk of age (20X magnification). Arrowheads indicate macrophages filling alveoli in lungs from M-TTP KO and M-triple KO mice. **(G, H, I)** H&E-stained sections of livers from (G) male control, (H) male M-TTP KO, and (I) male M-triple KO mice at 9 wk of age (20X magnification). Arrowheads indicate mononuclear cell infiltrates in livers from M-TTP KO and M-triple KO mice.

Complete necropsies were evaluated from control, M-triple KO and M-TTP KO mice, and a comparison between M-Triple KO, M-TTP KO, and control mice was performed. As shown above, spleen sizes in both male and female M-Triple KO mice were markedly increased compared with the spleen sizes from control mice (Fig 2F). Microscopic evaluation revealed moderate effacement of splenic red pulp by hematopoietic precursors, predominantly myeloid (myeloid hyperplasia) in variable stages of development, admixed with fewer foci of erythroid and megakaryocytic progenitors (Fig S2C). In addition, there were variable amounts of white pulp depletion, characterized by decreased lymphoid cellularity in periarteriolar lymphoid sheath size and number, decreased follicle size and/or number, and decreased marginal zone thickness in M-triple KO spleens (Fig S2C) when compared with spleens from control mice (Fig S2A). In contrast, spleens from M-TTP KO mice were only slightly larger than controls (Qiu et al, 2012), although microscopic evaluation revealed no splenic abnormalities (Fig S2B).

Lungs from M-triple KO mice revealed variable amounts of mixed cell inflammation, rare foamy macrophages (+/− eosinophilic spiculated material) and/or increased alveolar macrophages (Fig S2F) when compared with lungs from control mice (Fig S2D). Admixed were small numbers of multinucleated giant cells (macrophages), fewer eosinophils, and myeloid progenitors (myeloid hyperplasia) that expanded the alveolar interstitium and surrounded pulmonary vessels, and which rarely extended into the adjacent peri-pulmonary adipose tissue (Fig S2F). In contrast, male M-TTP KO mice had a minimal increase in alveolar macrophages; this finding was not observed in females (Fig S2E).

Livers from M-triple KO mice revealed perivascular, subcapsular, and/or sinusoidal myeloid cell progenitors (myeloid hyperplasia) (Fig S2I), admixed with multifocal mixed cell infiltrates that surrounded the vasculature (Fig S2I), when compared with livers from control mice (Fig S2G). In contrast, several liver sections from M-TTP KO mice contained minimal amounts of mixed cell inflammation (neutrophils and mononuclear cells) (Fig S2H).

Hearts from male and female M-triple KO mice contained variable amounts of neutrophils focally expanding the endocardial region, including heart valve and base (images not shown). In some M-triple KO animals, focal coronary vasculitis with adjacent myocardial necrosis, inflammation, and fibrosis was observed (images not shown). No pathological changes were observed in the hearts or aortas from 9-wk-old male and female M-TTP KO mice (images not shown).

Overall, complete necropsies of 7- to 10-wk-old male and female M-triple KO mice demonstrated neutrophilic inflammation and myeloid hyperplasia in multiple tissues, which was in stark contrast to the minimal inflammation observed in the skin (dermis), livers, and lungs of male and female M-TTP KO mice. These data suggest that knocking out *Zfp36l1* and *Zfp36l2*, in addition to *Zfp36*, in myeloid cells, leads to an increase in inflammatory processes in many tissues.

### Flow cytometry analysis of bone marrow

**Figure 5.**
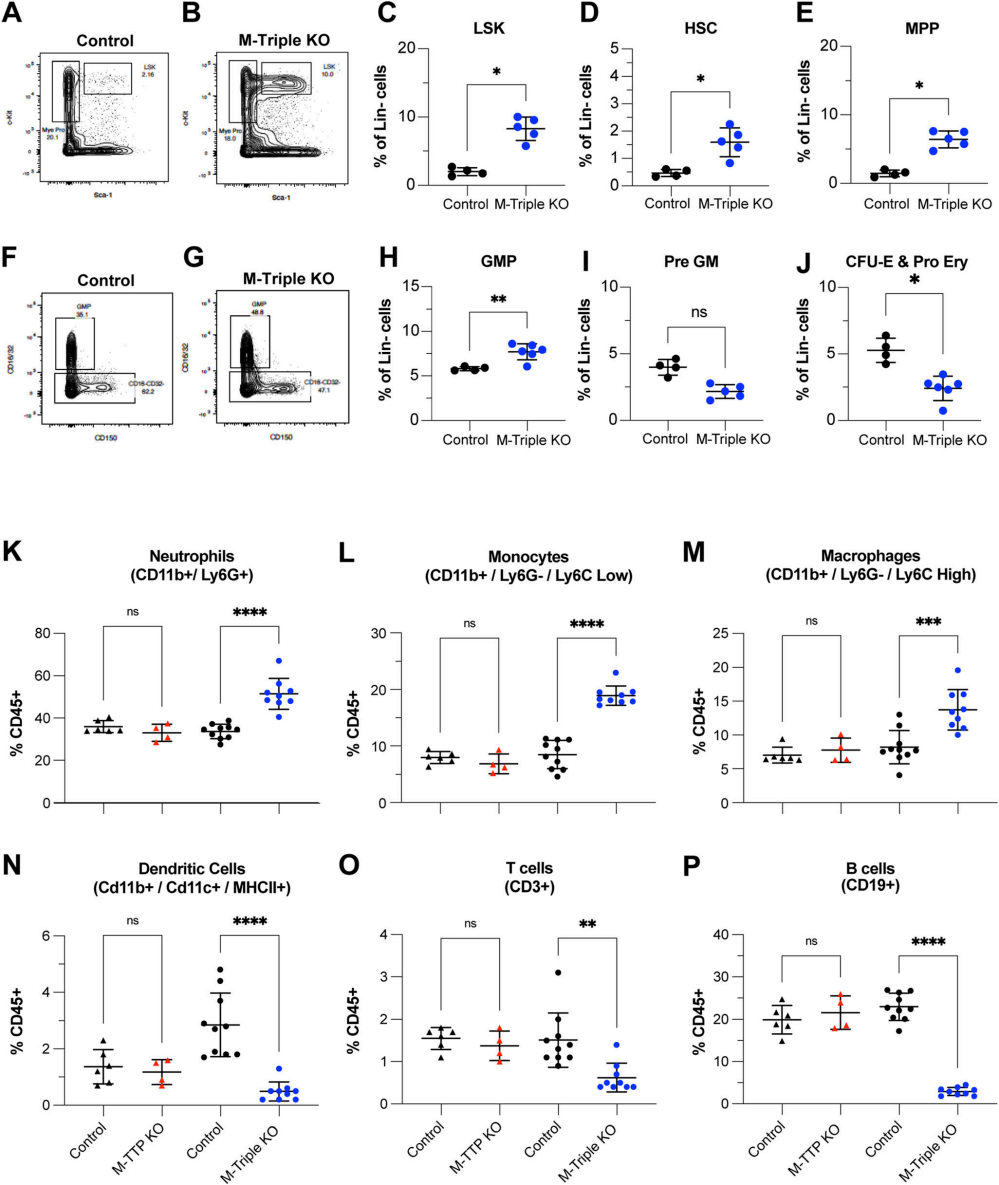
Effects of combined Zfp36 family member deficiency on myeloid cell populations in bone marrow. **(A, B, C, D, E, F, G, H, I, J, K, L, M, N, O, P)** Hematopoietic stem cell, myeloid cell progenitor, and mature myeloid cell populations in the bone marrow were quantitated by flow cytometry. **(A, B, F, G)** Shown are representative plots of flow cytometric analyses of the (A, B) LSK (Lin−, Sca1+, cKit+) and (F, G) granulocyte–macrophage progenitor (Lin−, Sca1−, c-Kit+, CD41−, CD16/32+) populations in total bone marrow from male and female 9-wk-old control (N = 4) and M-triple KO (N = 6) mice. The rectangular outlines indicate the gated population and the percentages of cells in that population. The following cell populations were quantitated in total bone marrow from male and female 9-wk-old control (N = 4) and M-triple KO (N = 6) mice, and shown as percentages of Lin- cells as means ± SD: **(C, D, E, H, I, J)** LSK (Lin−, Sca1+, cKit+), (D) Hematopoietic stem cell (Lin−, Sca1+, cKit+, CD150+), (E) multipotent progenitor population (Lin−, Sca1+, c-Kit+, CD150-), (H) granulocyte–macrophage progenitor (Lin−, Sca1−, cKit+, CD41−, CD16/32+), (I) pre-GM progenitor (Lin−, Sca1−, cKit+, CD41−, CD16/32−, CD150−, Endoglin−), and (J) colony forming unit-erythroid and pro-erythroid progenitors (Lin−, Sca1−, c-Kit+, CD41−, CD16/32−, CD150−, Endoglin+). *P < 0.05, determined by unpaired, two-tailed *t* tests with Welch’s correction. **(K, L, M, N, O, P)** The following mature myeloid and lymphoid cell populations were quantitated in total bone marrow from M-TTP KO (N = 4), M-TTP control (N = 6), M-Triple KO (N = 9), and control mice (N = 10) at 7–9 wk of age by flow cytometry, and shown as percentages of CD45^+^ cells as means ± SD: (K) neutrophils (CD45+/CD11b+/Ly6G+), (L) monocytes (CD45+/CD11b+/Ly6G-/Ly6C high), (M) macrophages (CD45+/CD11b+/Ly6G-/Ly6C low), (N) dendritic cells (CD45+/CD11b+/CD11c+/MHCII+), (O) T cells (Cd45+, CD3^+^), and (P) B cells (CD45^+^, CD19^+^). *P < 0.05; **P < 0.01; ***P < 0.001; ****P < 0.0001, determined by unpaired, two-tailed *t* tests with Welch’s correction.

To assess the hematopoietic stem cell (HSC) and myeloid progenitor populations in bone marrow, total bone marrow from control and M-triple KO mice at 8–10 wk of age was isolated and stained with a panel of antibodies. We combined data from both sexes for these analyses. There were significant increases in the Lin-/Sca-1+/cKit+ (LSK) population (Fig 5A–C), and the HSC (Fig 5D) and multipotent progenitor (Fig 5E) populations, in M-triple KO bone marrow when compared with controls. There was no change in the percentage of pre-granulocyte macrophage cells in M-triple KO bone marrow compared with controls (Fig 5I). However, the proportion of granulocyte–monocyte progenitors was increased in the M-triple KO bone marrow (Fig 5F–H), suggesting an increase in granulocyte, monocyte, and macrophage differentiation. The erythroid lineage fractions (pre-CFU-E and CFU-E) were also significantly decreased in the M-triple KO bone marrow (Fig 5J).

Bone marrow cells isolated from femurs of control, M-triple KO, and M-TTP KO mice at 8–10 wk of age were also stained with a panel of antibodies to quantitate the mature myeloid and lymphoid populations. Flow cytometry analyses of bone marrow cells from 8- to 10-wk-old M-TTP KO and control mice showed similar percentages of total myeloid cells, neutrophils, monocytes, macrophages, dendritic cells, T cells, and B cells (Fig 5K–P).

In contrast, flow cytometry analyses of bone marrow cells from M-triple KO mice demonstrated increased the percentages of the total myeloid cell population, and increased percentages of neutrophils, monocytes, and macrophages (Fig 5K–M), when compared with control bone marrow. In addition, M-triple KO bone marrow had decreased percentages of dendritic cells, T cells, and B cells (Fig 5N–P), when compared with control bone marrow. These data suggest that myeloid progenitors in the M-triple KO bone marrow were shifting towards granulocyte–macrophage differentiation over erythroid or lymphocyte differentiation. Overall, bone marrow from M-TTP KO mice exhibited normal proportions of myeloid and lymphoid cell populations, whereas bone marrow from M-triple KO mice had an overall increase in the myeloid cell populations and decreases in the lymphoid and erythroid populations.

### Peripheral blood analysis

**Figure 6.**
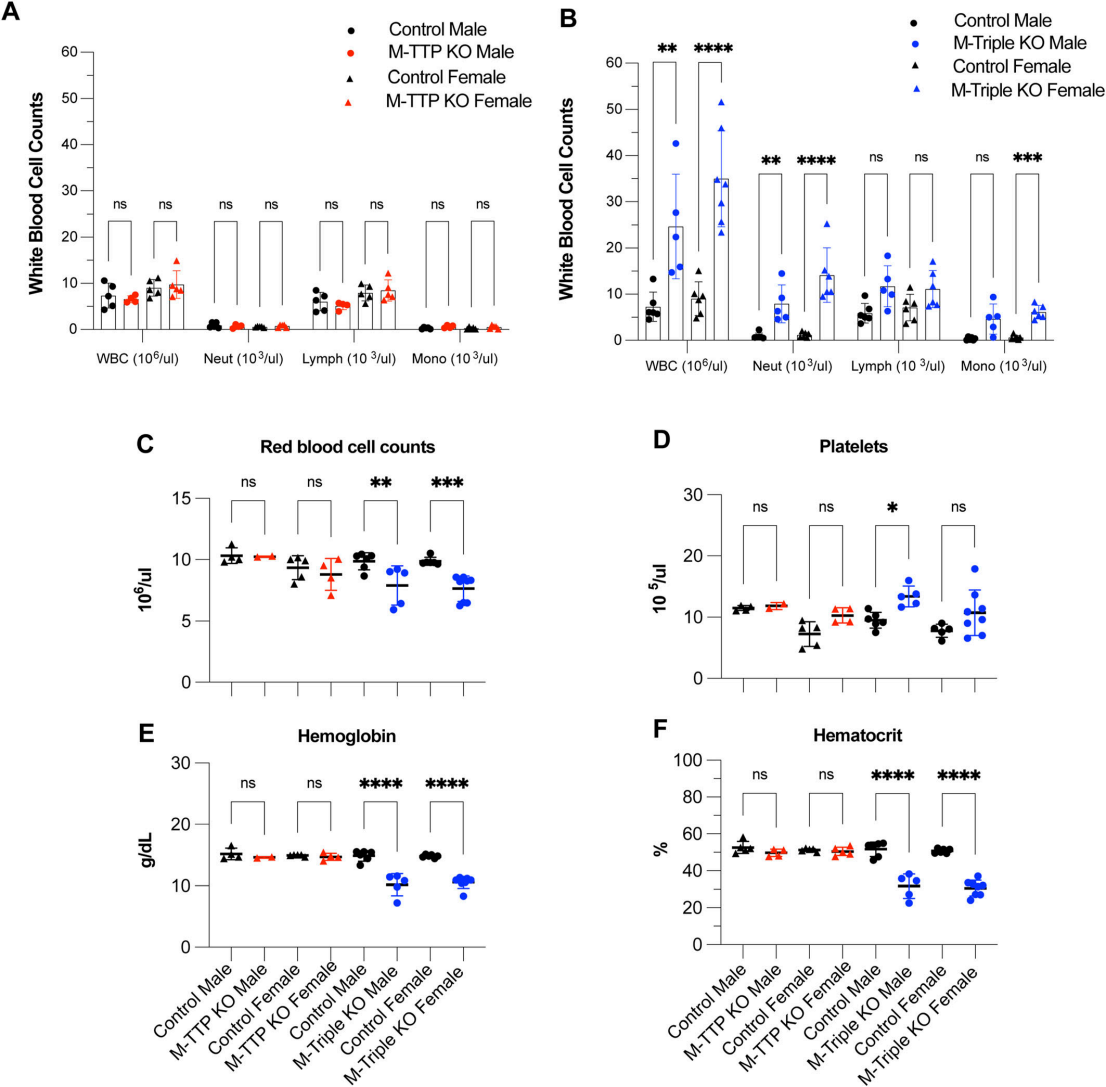
Peripheral blood analysis. Complete blood counts were performed on peripheral blood from both male and female M-TTP KO (N = 5), M-TTP control (N = 5), M-triple KO, and control mice (N = 6) at 7–9 wk of age. **(A, B)** The total white blood count and absolute white blood cell differential (neut: neutrophils; lymph: lymphocytes; mono: monocytes) counts in (A) M-TTP KO and M-TTP control and (B) M-triple KO and controls are shown as means ± SD. **(C, D, E, F)** The (C) total RBC, (D) platelets (Plts), (E) hemoglobin (Hgb) concentration, and (F) hematocrit (Hct) counts are shown as means ± SD. *P < 0.05; **P < 0.01; ***P < 0.001; ****P < 0.0001; determined by two-way ANOVA with Šídák’s multiple comparison test.

Analysis of peripheral blood at 8–10 wk of age revealed significant increases in the total white blood cell counts of male and female M-triple KO mice compared with control mice (Fig 6B), whereas there were no changes in the total white blood counts of male or female M-TTP KO mice compared with control mice (Fig 6A). The numbers of neutrophils were also significantly increased in the white blood cell differentials of peripheral blood from both male and female M-triple KO mice (Fig 6B). The numbers of monocytes were significantly increased in peripheral blood from female M-triple KO mice, but not from male M-triple KO mice, although the trend in male mice was in the same direction as in the females. There were no changes observed in the absolute white blood cell differentials of peripheral blood from M-TTP KO mice (Fig 6A), when compared with control mice.

The platelet counts were slightly increased in male M-triple KO mice compared with controls, but were not changed in peripheral blood from M-triple KO female, M-TTP KO male or M-TTP KO female mice (Fig 6D). The red blood cell count (Fig 6C), hemoglobin concentration (Fig 6E), and hematocrit (Fig 6F) were significantly decreased in male and female M-triple KO mice compared with control mice, whereas there were no changes in peripheral blood from male or female M-TTP KO mice compared with control mice (Fig 6). This also correlates with the decreased erythroid lineage fractions (pre-CFU-E and CFU-E) in the M-triple KO bone marrow (Fig 5J). These data demonstrate that knocking out *Zfp36l1* and *Zfp36l2* in addition to *Zfp36* in myeloid cells had a more global effect than knocking out *Zfp36* alone in these cells, and led to increased numbers of white blood cells in the peripheral blood.

### Serum cytokine analysis

**Figure 7.**
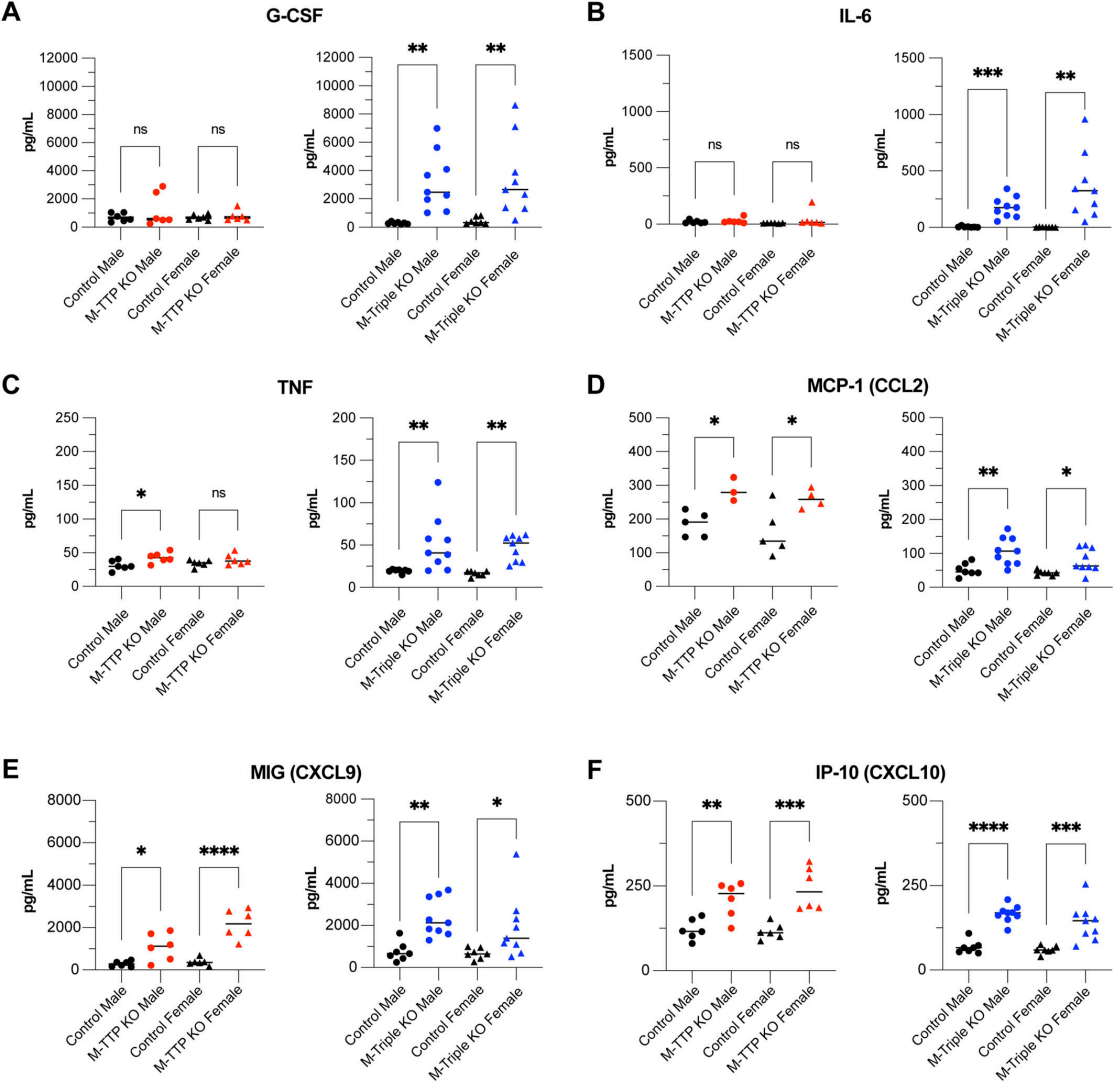
Cytokine analysis of serum from 9-wk-old male and female M-TTP control (N = 12), M-TTP KO (N = 12), M-triple KO (N = 17), and control (N = 14) mice. **(A, B, C, D, E, F)** Levels of (A) G-CSF, (B) IL-6, (C) TNF, (D) MCP-1 (CCL2), (E) MIG (CXCL9), and (F) IP-10 (CXCL10) were determined using plate-based cytokine immunoassays, and are shown as mean concentrations (pg/ml) ± SD. *P < 0.05; **P < 0.01; ***P < 0.001; ****P < 0.0001, using ordinary one-way ANOVA with Šídák’s multiple comparison test.

To determine whether cytokines and chemokines were hypersecreted into the blood of M-TTP KO or M-triple KO mice, we collected serum from controls, M-TTP KO, and M-triple KO mice and performed a 31-plex cytokine/chemokine analysis. Of the 31 cytokines/chemokines analyzed, we observed that six were significantly increased in the M-triple KO serum (Fig 7). G-CSF (Fig 7A), IL-6 (Fig 7B), and TNF (Fig 7C) were all significantly increased in the M-triple KO serum, but only TNF was slightly increased in the M-TTP KO serum (Fig 7C). CCL2 (Fig 7D), CXCL9 (Fig 7E), and CXCL10 (Fig 7F) were significantly increased in both the M-TTP KO and M-Triple KO serums, when compared with controls.

Overall, more cytokines and chemokines were elevated in the M-triple KO serum than in M-TTP KO serum, when compared with control serum. Many of the increased cytokines and chemokines are associated with chemotaxis and recruitment of leukocytes to sites of inflammation. In addition, four of the six cytokines/chemokines that were elevated in the M-Triple KO serum are encoded by mRNAs that are thought to be direct TTP targets: *Csf3* (Brooks & Blackshear, 2013), *Tnf* (Carballo et al, 1998; Lai et al, 1999), *Il6* (Sauer et al, 2006; Zhao et al, 2011), and *Ccl2* (Sauer et al, 2006). In short, knocking out *Zfp36*, *Zfp36l1*, and *Zfp36l2* simultaneously in myeloid cells had a greater effect on serum cytokine expression than myeloid-specific *Zfp36*-KO alone.

### RNA-seq analysis of differentially expressed genes (DEGs) in primary BMDM

To identify differential gene expression changes in M-triple KO macrophages compared with control and M-TTP KO macrophages, we cultured BMDM from 9-wk-old control, M-TTP KO, and M-Triple KO mice, and measured mRNA levels and decay rates by RNA-seq before and after 1 h of LPS treatment, followed by addition of actinomycin D (ActD). After quality control and alignment of sequence reads to the transcriptome, relative expression levels were calculated and expressed as normalized reads per kilobase. We evaluated only mRNAs and ignored transcripts with mean expression levels below 0.1 FPKM at time 0 in the control samples (equivalent to 5.8 normalized reads per kilobase in the M-triple KO experiment and 6.4 normalized reads per kilobase in the M-TTP KO experiment). We also removed transcripts from one of the knocked-out genes (*Zfp36*) and its artefactually elevated downstream gene, *Plekhg2* (data not shown). We used DESeq2 to identify DEGs using the criteria of a log_2_ fold change ≥ 0.3785 (or a raw fold change of 1.3) for M-TTP KO or M-triple KO compared with controls, and an adjusted *P*-value ≤ 0.05. Once DEGs were identified, we used a custom search application to identify which transcripts contained one or more potential TTP binding sites, as previously described (Lai et al, 2013). We compared the list of DEGs with lists of previously identified TTP targets (Brooks & Blackshear, 2013; Patial et al, 2016b). The transcripts that were significantly up-regulated and down-regulated in the M-TTP KO and M-triple KO macrophages before and after LPS are listed in Tables S1, S2, S3, and S4. In addition, the transcripts up- or down-regulated in both the M-TTP KO and M-triple KO macrophages before and after LPS are listed in Tables S5 and S6, respectively.

Table S1. Up- and down-regulated transcripts in M-TTP KO BMDM before LPS treatment. This table contains seven transcripts that were significantly up- or down-regulated in the M-TTP KO BMDM in comparison with control BMDM before LPS treatment, using the criteria of a log_2_ fold change ≥ 0.3785 (or a raw fold change of 1.3) and an adjusted *P*-value ≤ 0.05 using DESeq2. Column A indicates the gene symbol. Column B indicates whether the listed transcripts contained one or more potential TTP binding sites in the 3′-UTR. Column C indicates whether the designated transcripts have been previously identified as known or suspected TTP targets in the current literature. Columns D and E indicated whether the listed transcripts were significantly up- or down-regulated by a log_2_ fold change ≥ 0.3785 (or a raw fold change of 1.3) and an adjusted *P*-value ≤ 0.05 in the M-TTP KO or M-triple KO BMDM, respectively, in comparison with controls as determined by DESeq2. Columns F-I indicate the expression levels of the designated transcripts, shown as mean normalized counts per kilobase for each designated genotype and time point. Columns J and K indicate the log_2_ fold change in the average values for the listed transcripts in M-TTP KO or M-triple KO BMDM before LPS treatment, in comparison with controls. Columns L and M indicate the adjusted *P*-values (*P*adj) of M-TTP KO or M-triple KO BMDM before LPS treatment, in comparison to controls. Transcripts that were significantly up-regulated are shown in black, and transcripts that were significantly down-regulated are shown in blue. Transcripts shown in bold were also significantly up-regulated in the M-triple KO macrophages in comparison with controls (Sheet 3).

Table S2. Up- and down-regulated transcripts in M-TTP KO BMDM after 1 h of 1 μg/ml LPS treatment. This table contains 20 transcripts that were significantly up- or down-regulated in M-TTP KO BMDM in comparison with control BMDM after 1 h of 1 μg/ml LPS treatment, using the criteria of a log_2_ fold change ≥ 0.3785 (or a raw fold change of 1.3) and an adjusted *P*-value ≤ 0.05 using DESeq2. Column A indicates the gene symbol. Column B indicates whether the listed transcripts contained one or more potential TTP binding sites in the 3′-UTR. Column C indicates whether the listed transcripts have been previously identified as known or suspected TTP targets in the current literature. Columns D and E indicate whether the listed transcripts were significantly up- or down-regulated by a log_2_ fold change ≥ 0.3785 (or a raw fold change of 1.3), and an adjusted *P*-value ≤ 0.05, in the M-TTP KO or M-triple KO BMDM, respectively, in comparison with controls, as determined by DESeq2 statistics. Columns F–I indicate the expression levels of the designated transcripts shown as mean normalized counts per kilobase for each designated genotype and time point. Columns J and K indicate the log_2_ fold changes of M-TTP KO or M-Triple KO BMDM before LPS treatment, in comparison to controls. Columns L and M show the adjusted *P*-values (*P*adj) of M-TTP KO or M-triple KO BMDM before LPS treatment, in comparison with controls. Transcripts that were significantly up-regulated are shown in black and transcripts that were significantly down-regulated are shown in blue. Transcripts shown in bold were also significantly up-regulated in the M-triple KO macrophages in comparison with controls.

Table S3. Up- and down-regulated transcripts in M-triple KO BMDM before LPS treatment. This table contains 2,638 transcripts that were significantly up- or down-regulated in the M-triple KO BMDM in comparison with control BMDM before LPS treatment, using the criteria of a log_2_ fold change ≥ 0.3785 (or a raw fold change of 1.3) and an adjusted *P*-value ≤ 0.05 using DESeq2. Column A indicates the gene symbol. Column B indicates whether the listed transcript contained one or more potential TTP binding sites in its 3′-UTR. Column C indicates whether the listed transcripts have been previously identified as known or suspected TTP targets in the current literature. Columns D and E indicate the expression levels of the designated transcripts, shown as mean normalized counts per kilobase for each designated genotype and time point. Column F indicates the log_2_ fold change of mean transcript levels from M-triple KO BMDM before LPS treatment, in comparison with control means. Column G shows the adjusted *P*-values (*P*adj) of mean transcript levels from M-triple KO BMDM before LPS treatment, in comparison with controls. Transcripts that were significantly up-regulated are shown in black and transcripts that were significantly down-regulated are shown in blue.

Table S4. Up- and down-regulated transcripts in M-Triple KO BMDM after 1 h of 1 μg/ml LPS treatment. This table contains 2,063 transcripts that were significantly up- or down-regulated in the M-triple KO BMDM in comparison with control BMDM after 1 h of 1 μg/ml LPS treatment, using the criteria of a log_2_ fold change ≥ 0.3785 (or a raw fold change of 1.3) and an adjusted *P*-value ≤ 0.05 using DESeq2. Column A indicates the gene symbol. Column B indicates whether the listed transcript contained one or more potential TTP binding sites in its 3′-UTR. Column C indicates whether the listed transcripts have been previously identified as a known or suspected TTP target in the current literature. Columns D and E indicate the expression levels of the designated transcripts, shown as mean normalized count per kilobase for each designated genotype and time point. Column F indicates the log_2_ fold change of mean transcript levels from M-triple KO BMDM after 1 h of 1 μg/ml LPS treatment, in comparison with controls. Column G shows the adjusted *P*-values (*P*adj) of transcript levels from M-triple KO BMDM after 1 h of 1 μg/ml LPS treatment, in comparison with controls. Transcripts that were significantly up-regulated are shown in black and transcripts that were significantly down-regulated are shown in blue.

Table S5. Transcripts that were up- or down-regulated in both the M-TTP KO and M-triple KO BMDM before LPS treatment. This table contains two transcripts that were significantly up- or down-regulated in both the M-TTP KO and M-triple KO BMDM in comparison with control BMDM before LPS treatment, using the criteria of a log_2_ fold change ≥ 0.3785 (or a raw fold change of 1.3) and an adjusted *P*-value ≤ 0.05 using DESeq2. Column A indicates the gene symbol. Column B indicates whether the listed transcripts contained one or more potential TTP binding sites in the 3′-UTR. Column C indicates whether the designated transcripts have been previously identified as known or suspected TTP targets in the current literature. Columns D and E indicate whether the listed transcripts were significantly up- or down-regulated by a log_2_ fold change ≥ 0.3785 (or a raw fold change of 1.3) and an adjusted *P*-value ≤ 0.05 in the M-TTP KO or M-triple KO BMDM, respectively, in comparison with controls, as determined by DESeq2 statistics. Columns F–I indicate the expression level of the designated transcripts shown as mean normalized count per Kb for each designated genotype and time point. Columns J and K indicate the log_2_ fold change in expression of the listed transcripts in M-TTP KO or M-triple KO BMDM before LPS treatment, in comparison with controls. Columns L and M indicate the adjusted *P*-values (*P*adj) when comparing mean transcript levels from M-TTP KO or M-triple KO BMDM before LPS treatment, in comparison with controls. Transcripts that were significantly up-regulated are shown in black and transcripts that were significantly down-regulated are shown in blue.

Table S6. Transcripts that were up- or down-regulated in both M-TTP KO and M-triple KO BMDM after 1 h of 1 μg/ml LPS treatment. This table contains 11 transcripts that were significantly up- or down-regulated in both the M-TTP KO and M-triple KO BMDM, in comparison with control BMDM, after 1 h of 1 μg/ml LPS treatment, using the criteria of a log_2_ fold change ≥ 0.3785 (or a raw fold change of 1.3) and an adjusted *P*-value ≤ 0.05, using DESeq2. Column A indicates the gene symbol. Column B indicates whether the listed transcripts contained one or more potential TTP binding site in the 3′-UTR. Column C indicates whether the designated transcripts have been previously identified as known or suspected TTP targets in the current literature. Columns D and E indicate whether the listed transcripts were significantly up- or down-regulated by a log_2_ fold change ≥ 0.3785 (or a raw fold change of 1.3) and an adjusted *P*-value ≤ 0.05 in the M-TTP KO or M-Triple KO BMDM, respectively, in comparison with controls, as determined by DESeq2 statistics. Columns F through I indicate the expression levels of the designated transcripts, shown as mean normalized counts per kilobase for each designated genotype and time point. Columns J and K indicate the log_2_ fold changes in mean levels of expression of the listed transcripts in M-TTP KO or M-triple KO BMDM after 1 h of 1 μg/ml LPS treatment, in comparison with controls. Columns L and M indicate the adjusted *P*-values (*P*adj) of mean transcript levels from M-TTP KO or M-triple KO BMDM after 1 h of 1 μg/ml LPS treatment, in comparison with controls. Transcripts that were significantly up-regulated are shown in black, and transcripts that were significantly down-regulated are shown in blue.

**Figure 8.**
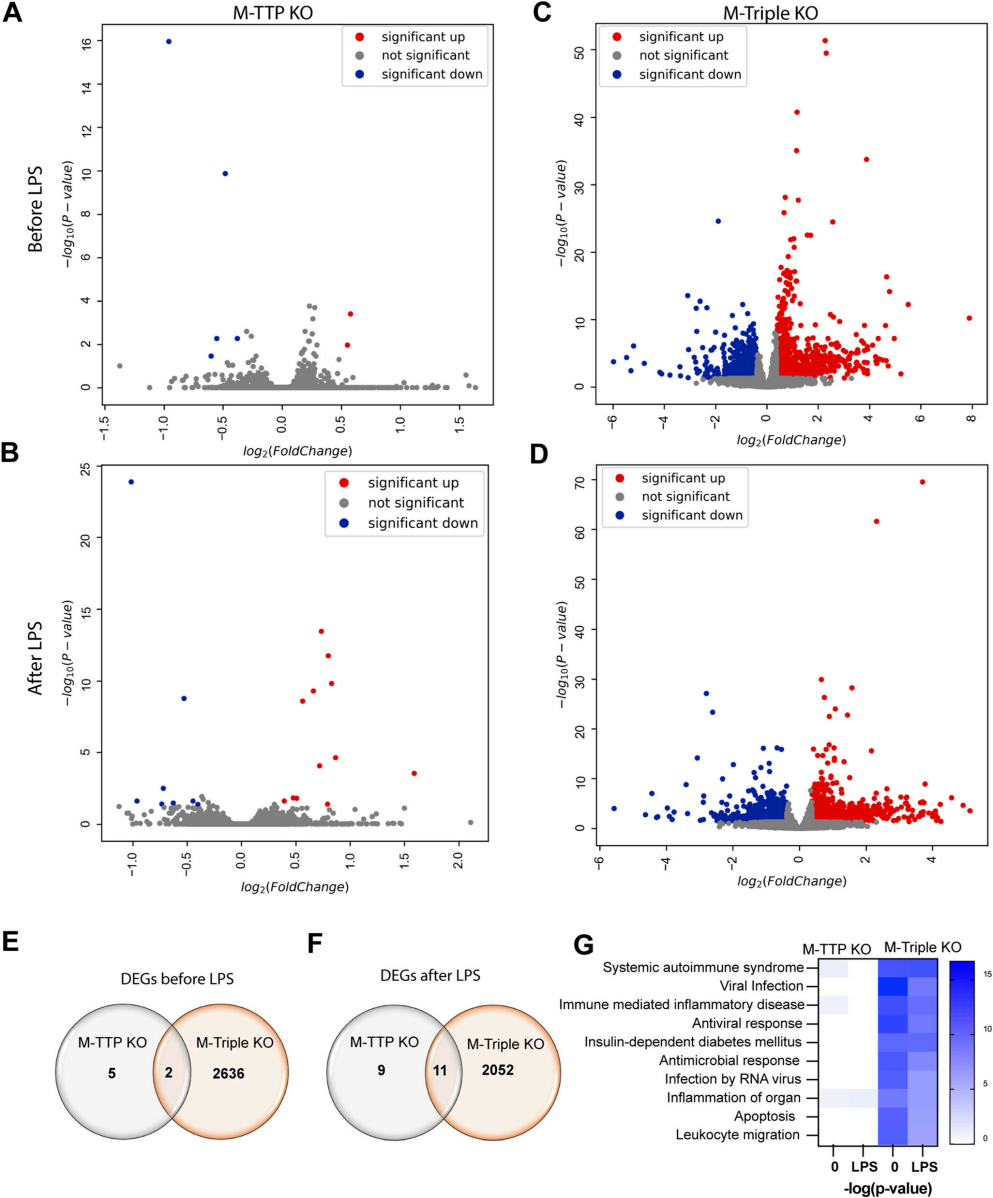
RNA-seq analysis of differentially regulated transcripts in BMDM. BMDM from 9-wk-old male controls, M-TTP KO, and M-triple KO mice were treated with or without 1 μg/ml LPS for 1 h. **(A, B, C, D)** Volcano plots showing differentially expressed genes in BMDM from controls, M-TTP KO, and M-triple KO mice before (A, B) and after 1 h of LPS treatment (C, D). Significantly up-regulated transcripts whose means were at least 1.3fold above control values are shown in red, whereas the transcripts down-regulated to a comparable extent are shown in blue. Transcripts with no significant change between control and KO are shown in grey. **(E, F)** Venn diagrams demonstrate the overlap in the numbers of transcripts up- or down-regulated in M-TTP KO compared with M-triple KO cells (E) before and (F) after LPS treatment. **(G)** Heatmaps are shown indicating the top 10 involved pathways, with white showing no change and blue showing a highly significant *P*-value for a given disease and biological function enriched in TTP KO and triple KO macrophages, compared with controls before and after LPS treatment, determined by Ingenuity Pathway Analysis.

In M-TTP KO macrophages before LPS treatment, two transcripts were up-regulated by 1.3fold or more (*Asb1* and *Cebpb* mRNAs), of which only *Asb1 mRNA* had one or more potential TTP-binding sites (Fig 8A and Table S1). Five transcripts were 1.3fold down-regulated in the M-TTP KO macrophages (*Fam135a*, *Zranb3*, *Chst3*, *Arhgap32*, and *Lyz2*), of which, *Fam135a* and *Chst3* mRNAs had one or more potential TTP binding sites (Fig 8A and Table S1). After LPS treatment, 12 transcripts were significantly more than 1.3fold up-regulated in the M-TTP KO macrophages (*Rab3a*, *Cxcl2*, *Asb1*, *Mllt11*, *Ier3*, *Tnf*, *Tmcc2*, *Zfp36l2*, *B4galt3*, *Gadd45a*, *Mdm1*, and *Yrdc* mRNAs), all of which contained one or more potential TTP binding sites (Fig 8B and Table S2). 4 of the 12 up-regulated transcripts (*Cxcl2*, *Ier3*, *Tnf*, and *Tmcc2* mRNAs) have been previously identified as demonstrated or proposed TTP targets (Brooks & Blackshear, 2013). After LPS treatment, eight transcripts were 1.3fold down-regulated (*2510009E07Rik*, *Pole*, *Zranb3*, *Bsn*, *Chst3*, *Itgax*, *Eda2r*, and *Lyz2* mRNAs) in the M-TTP KO macrophages, and two of these (*Chst3* and *Eda2r* mRNAs) contained one or more potential TTP binding sites (Fig 8B and Table S2).

In the M-triple KO macrophages before LPS treatment, 1,415 transcripts were significantly up-regulated by 1.3fold or more, and 1,223 transcripts were significantly down-regulated by 1.3fold or more, when compared with control macrophages (Fig 8C and Table S3). Of the 1,415 up-regulated transcripts, 607 (43%) contained one or more potential TTP binding sites, whereas 337 of the 1,223 (28%) down-regulated transcripts contained one or more potential TTP binding sites (Table S3). Seventeen of the up-regulated transcripts (*Ccl2*, *Nos2*, Ccl3, *Il10*, *Serpinb2*, *Ier3*, *Il1β*, *Tnf*, *Pim1*, *Cxcl1*, *Plaur*, *Cxcl2*, *Cdkn1a*, *Tlr4I*, *Ube3a*, *Lats2*, and *E2f1* mRNAs) and five of the down-regulated transcripts (*Tcf3*, *Myc*, *Cxcr4*, *Clmp*, and *Vegfa mRNAs*) were previously identified as demonstrated or proposed TTP targets (Table S3) (Brooks & Blackshear, 2013). After LPS treatment, 1,089 transcripts were significantly up-regulated by more than 1.3fold, and 974 transcripts were 1.3fold or more down-regulated, in the M-triple KO macrophages compared with control macrophages (Fig 8D and Table S4). 420 of the 1,089 (39%) up-regulated transcripts and 308 of the 974 (32%) down-regulated transcripts in the M-triple KO macrophages contained one or more potential TTP binding sites (Table S4). Six of the up-regulated transcripts (*Thbd*, *Ier3*, *Tnf*, *Tlr4*, *E2f1*, and *Ube3a*) and four of the down-regulated transcripts (*Tcf3*, *Ptgs2*, *Clmp*, and *Vegfa*) in the M-triple KO macrophages were previously identified as demonstrated or proposed TTP targets (Table S4) (Brooks & Blackshear, 2013). Interestingly, more transcripts were significantly changed in the M-triple KO macrophages before LPS treatment than after LPS treatment (Fig 8).

Before LPS treatment, *Asb1* mRNA was the only transcript that was more than 1.3fold up-regulated, and *Lyz2* mRNA was the only transcript that was more than 1.3fold down-regulated in both the M-TTP KO and M-Triple KO macrophages compared with control cells (Fig 8E and Table S5). After LPS treatment, eight transcripts (*Rab3a*, *Asb1*, *Mllt11*, *Ier3*, *Tnf*, *Tmcc2*, *B4galt3*, and *Mdm1*) were up-regulated in both the M-TTP KO and M-triple KO macrophages, and all contained one or more potential TTP binding sites (Fig 8F and Table S6). After LPS treatment, three transcripts (*Itgax*, *Eda2r*, and *Lyz2* mRNAs) were significantly down-regulated in both the M-TTP KO and M-triple KO macrophages (Fig 8F and Table S6). One (*Eda2r*) of the three down-regulated transcripts contained one or more TTP binding sites (Table S6). In both the untreated (Fig 8E) and LPS-treated (Fig 8F) conditions, there were many more differentially regulated transcripts in the M-triple KO macrophages than there were in the M-TTP KO macrophages. Ingenuity pathway analysis (IPA) revealed gene enrichment associated with many diseases, such as systemic autoimmune syndrome, viral infection, and immune-mediated inflammatory diseases in the M-triple KO macrophages before and after LPS, but not in the M-TTP KO macrophages (Fig 8G).

### RNA-seq analysis of mRNA decay

We also used RNA-seq to measure mRNA decay on a genome-wide scale in control, M-TTP KO, and M-triple KO macrophages after 1 h of LPS treatment, followed by addition of ActD. To determine differences in mRNA decay, we calculated the percentage of the original mRNA remaining after the 1 h LPS time point, after removing non-mRNAs, the knocked out genes (*Zfp36*, *Zfp36l1*, and *Zfp36l2*), the artefactually up-regulated gene *Plekhg2*, and mRNAs whose mean levels in the control cells were <0.1 FPKM. We also removed any transcripts that did not decrease to below 85% mRNA remaining in control BMDM after 120 min of ActD. After these initial cutoffs, 4,167 and 4,572 transcripts remained in the M-triple KO and M-TTP KO datasets, respectively. Because we had a large number of decay curves to analyze, we completed an initial screen to select transcripts that appeared to be significantly stabilized and exhibited *P*-values < 0.008, as determined by unpaired two-tailed *t* tests with Bonferroni correction between the control and M-TTP KO cells (Table S7) or the control and M-triple KO cells (Table S8) at three consecutive time-points. From the transcript list created by this initial screen, we performed two-way ANOVA with Geisser–Greenhouse correction and Šídák’s multiple comparison test as a more stringent statistical test of the entire decay curve, and removed any transcripts with an ANOVA *P*-value > 0.05. The apparently stabilized transcripts were then ranked by the biggest difference in the average percent mRNA remaining at the 60- and 120-min time points between the control and M-TTP KO samples, or the control and M-triple KO samples. One caveat is that addition of ActD is associated with the decay of many transcripts, resulting in a decrease of the total RNA isolated at later time points. Standard normalization methods used in RNA-seq assume that libraries were prepared from comparable pools of RNA. Therefore, levels of non-decaying or slowly decaying transcripts will artefactually appear to increase over time (Lai et al, 2019a). However, in the experiments described below, each pair of WT and KO cells was treated in the same experiment, and therefore, any apparent drift in the baselines should be comparable between the genotypes throughout the experiment.

Table S7. Transcripts with significantly delayed mRNA decay at three consecutive time points in M-TTP KO BMDM compared with control BMDM. RNA-seq was used to measure mRNA decay differences on a genome-wide scale in control and M-TTP KO BMDM after 1 h of LPS treatment, followed by ActD treatment. To determine differences in mRNA decay, we calculated the percentage of the original mRNA remaining after the 1 h LPS time point, and selected transcripts that had statistically significant changes in average mRNA levels at a given time point. This table contains 15 transcripts with significantly delayed mRNA decay in M-TTP KO BMDM after LPS and ActD treatment, when compared with control BMDM under the same conditions. Data are from macrophages derived from four mice in each group, and each time point used RNA from three plates of cells that were pooled. Column A indicates the gene symbol. Column B indicates whether the listed transcripts contained one or more potential TTP binding sites in the 3′-UTR. Column C indicates whether the designated transcripts have been previously identified as known or suspected TTP targets in the current literature. In an initial screen, we selected transcripts whose means were significantly different between the control and M-TTP KO cells after LPS and ActD treatment, with *P*-values < 0.0008, as determined by unpaired two-tailed *t* tests with Bonferroni correction. Columns E through H indicate whether there was a significant difference in the percentage of mRNA remaining in the M-TTP KO cells for three consecutive time points, as indicated, when compared with control cells. Columns J through O indicate the *P*-values between the M-TTP KO and control cells at the indicated time points, as determined by unpaired two-tailed *t* tests with Bonferroni correction. From this initial list, we also used the two-way analysis of variance (ANOVA) with Šídák’s multiple comparison test to determine how the mRNA decay of a given transcript was affected by two factors: (1) genotype and (2) treatment with LPS followed by the ActD time course. We considered there to be a significant difference in the decay of a given transcript between the two genotypes after these treatments, when the two-way ANOVA *P*-value was < 0.05. Column Q indicates whether there was a significant *P*-value in M-TTP KO BMDM compared with controls when using the two-way ANOVA with Šídák’s multiple comparison test, and column R indicates the respective *P*-value using this test. Column S indicates whether there was also a significant delay in mRNA decay in the M-triple KO cells in comparison with controls for the indicated transcripts that were stabilized in the M-TTP KO cells. Columns U through AA indicate the average percentage of the original mRNA remaining in control BMDM after LPS treatment, followed by the designated time point of ActD treatment. Columns AC through AH indicate the standard deviations of the percentage of mRNA remaining at each time point in control cells after LPS and ActD treatment. Columns AJ through AP indicate the average percentages of the original mRNA remaining in M-TTP KO BMDM after LPS treatment, followed by the designated time point of ActD treatment. Columns AR through AW indicate the standard deviations of the percentage of mRNA remaining at each time point in M-TTP KO cells after LPS and ActD treatment.

Table S8. Transcripts with significantly delayed mRNA decay at three consecutive time points in M-triple KO BMDM compared with control BMDM. RNA-seq was used to measure mRNA decay differences on a genome-wide scale in control and M-triple KO BMDM after 1 h of LPS treatment, followed by various time points of ActD treatment. To determine differences in mRNA decay, we calculated the percentage of the original mRNA remaining after the 1 h LPS time point and selected transcripts that had statistically significant changes in decay. This table contains 71 transcripts with significantly delayed mRNA decay in M-triple KO BMDM after LPS and ActD treatment, when compared with control BMDM under the same conditions. Data are from macrophages derived from four mice in each group, and each time point used three plates of cells that were pooled. Column A indicates the gene symbol. Column B indicates whether the listed transcripts contained one or more potential TTP binding sites in the 3′-UTR. Column C indicates whether the designated transcripts have been previously identified as known or suspected TTP targets in the current literature. In an initial screen, we selected transcripts that were significantly stabilized, with *P*-values < 0.0008, as determined by unpaired two-tailed *t* tests with Bonferroni correction between the control and M-triple KO cells after LPS and ActD treatment. Columns E through H indicate whether there were significant differences in the percentages of mRNA remaining in the M-triple KO cells for three consecutive time points as indicated, when compared with control cells. Columns J through O indicate the *P*-values between the M-triple KO and control cells at the indicated time points, as determined by unpaired two-tailed *t* tests with Bonferroni correction. From this initial list, we also used the two-way analysis of variance (ANOVA) with Šídák’s multiple comparison test to determine how the mRNA decay of a given transcript was affected by two factors: (1) genotype and (2) treatment with LPS followed by the ActD time course. We considered there to be a significant difference in the decay of a given transcript between the two genotypes after these treatments, when the two-way ANOVA *P*-value was < 0.05. Column Q indicates whether there was a significant *P*-value in M-triple KO BMDM compared with controls when using the two-way ANOVA with Šídák’s multiple comparison test, and column R indicates the respective *P*-value using this test. Columns T through Z indicate the average percentage of the original mRNA remaining in control BMDM after LPS treatment, followed by the designated time point of ActD treatment. Columns AB through AG indicate the SD of the percentage of mRNA remaining at each time point in control cells after LPS and ActD treatment. Columns AI through AO indicate the average percentage of the original mRNA remaining in M-triple KO BMDM after LPS treatment and the designated time point of ActD treatment. Columns AQ through AV indicate the SD of the percentage of mRNA remaining at each time point in M-triple KO cells after LPS and ActD treatment.

**Figure 9.**
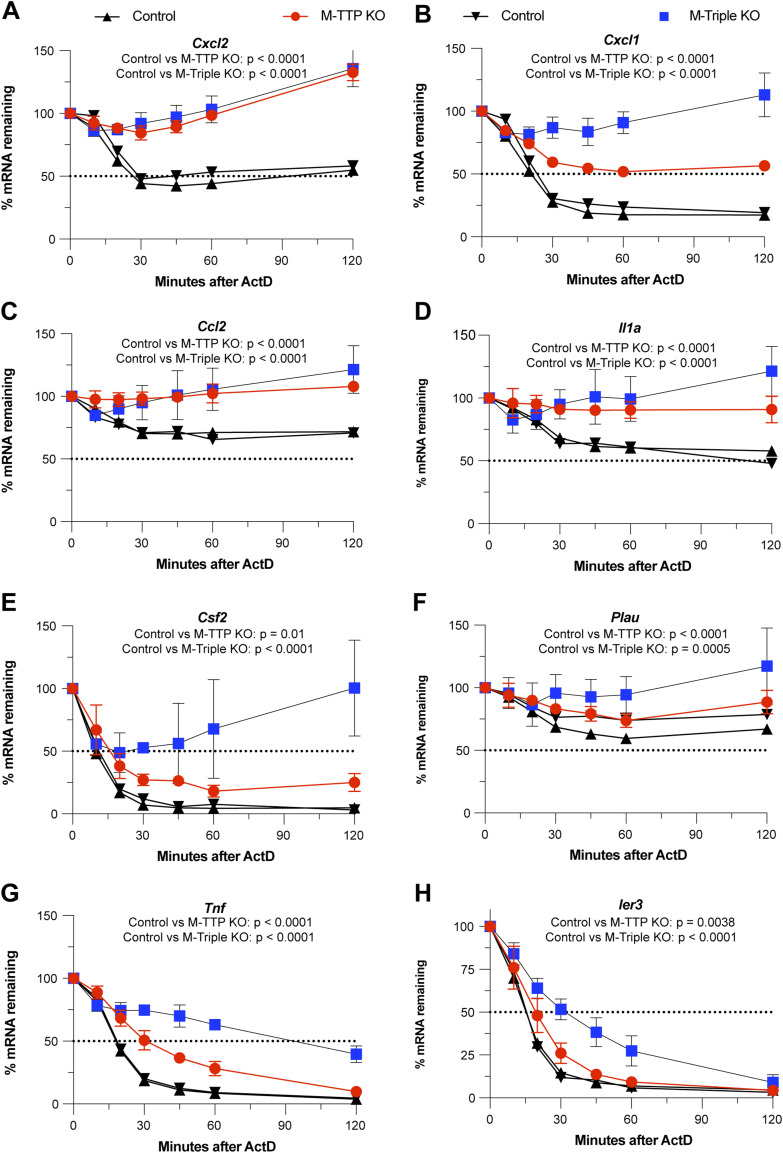
RNA-seq analysis of mRNA decay of TTP targets in BMDM. BMDM from male 9-wk-old control, M-TTP KO, and M-triple KO mice were cultured and treated with 1 μg/ml LPS for 1 h, followed by treatment with 5 μg/ml actinomycin D for various time points (cells were cultured from four animals in each group). Eight transcripts that have been previously identified or proposed as TTP targets were statistically significantly stabilized at three consecutive time points in the M-TTP KO macrophages compared with controls, as determined by unpaired two-tailed *t* tests. **(A, B, C, D, E, F, G)** Decay curves of (A) *Cxcl2*, (B) *Cxcl1*, (C) *Ccl2*, (D) *Il1a*, (E) *Csf2*, (F) *Plau*, (G) *Tnf*, and (H) *Ier3* mRNAs in M-TTP KO and M-triple KO BMDM compared with control BMDM are shown as the percentages of mRNA remaining after 1 h of LPS treatment ± SD over 120 min after actinomycin D treatment. *P*-values are reported under the gene names as determined by repeated measures (RM) two-way ANOVA with the Geisser–Greenhouse correction and Šídák’s multiple comparison test.

15 transcripts were significantly stabilized after ActD treatment in the M-TTP KO macrophages compared with the controls using these criteria (Table S7). 13 of the 15 (87%) transcripts contained one or more characteristic TTP binding sites, and 8 of the 15 (53%) transcripts (*Cxcl2*, *Cxcl1*, *Ccl2*, *Il1α*, *Csf2*, *Plau*, *Tnf*, and *Ier3*) have been previously identified as known or suggested TTP targets (Fig 9 and Table S7) (Brooks & Blackshear, 2013). 9 of these 15 (60%) transcripts (*Cxcl2*, *Gdf15*, *Cxcl1*, *Ccl2*, *Il1α*, *Tnf*, *B4galt3*, *Tnfsf9*, and *Ier3*) were also significantly increased at these time points in the M-triple KO macrophages when compared with controls (Table S7).

Some mRNAs, such as *Cxcl2* (Fig 9A), *Ccl2* (Fig 9C), *Il1α* (Fig 9D), and *Plau* (Fig 9F), were significantly stabilized to similar extents in both the M-TTP KO and M-triple KO macrophages when compared with control macrophages (Fig 9). For example, the half-life of *Cxcl2* mRNA was ∼20 min in control macrophages; however, it was greater than 120 min in both M-TTP KO and M-triple KO macrophages (Fig 9A). In other examples, transcripts such as *Cxcl1* (Fig 9B), *Csf2* (Fig 9E), *Tnf* (Fig 9G), and *Ier3* (Fig 9H) were stabilized to a greater extent in the M-triple KO macrophages than in the M-TTP KO cells (Fig 9). In the case of *Cxcl1*, the half-life of the mRNA increased from ∼20 min in controls to more than 60 min in the M-TTP KO cells, but transcript levels remained above 100%, even after 120 min of ActD treatment, in the M-triple KO cells (Fig 9B). The half-life of *Tnf* mRNA was roughly 20 min in control macrophages, but it was only slightly greater in M-TTP KO macrophages, to about 30 min (Fig 9G). However, in M-triple KO macrophages, the *Tnf* mRNA half-life was greater than 100 min (Fig 9G). In this case, TTP on its own appears to regulate *Tnf* and *Cxcl1* mRNA stability to a relatively minor extent, but additional depletion of ZFP36L1 and ZFP36L2 leads to a greatly increased effect on *Tnf* and *Cxcl1* mRNA stability.

In the M-triple KO macrophages, 71 transcripts were significantly stabilized using these criteria when compared with control cells (Table S8). 58 of the 71 (82%) significantly stabilized transcripts contained one or more characteristic TTP binding sites, and 9 of the 71 (*Cxcl1*, *Cxcl2*, *Il1α*, *Tnf*, *Ccl2*, *Plk3*, *Pim1*, *Ier3*, and *Cxcr4*) have been previously identified as known or suggested TTP targets (Table S8). The top eight transcripts stabilized in the M-triple KO macrophages, in order of the biggest average differences between the control and M-triple KO last two time-points were *Rab3a*, *Cxcl1*, *Cxcl2*, *Gdf15*, *Il1α*, *Tnf*, *Hbegf*, and *Osm* (Table S8). Of the top transcripts, *Cxcl1*, *Cxcl2*, and *Tnf* are well-established TTP target mRNAs and have been previously shown to be stabilized in TTP KO fibroblasts and macrophages (Lai et al, 2006, 2018, 2019b). *Ccl2* and *Ccl3* have also been previously identified as likely TTP targets (Brooks & Blackshear, 2013).

**Figure 10.**
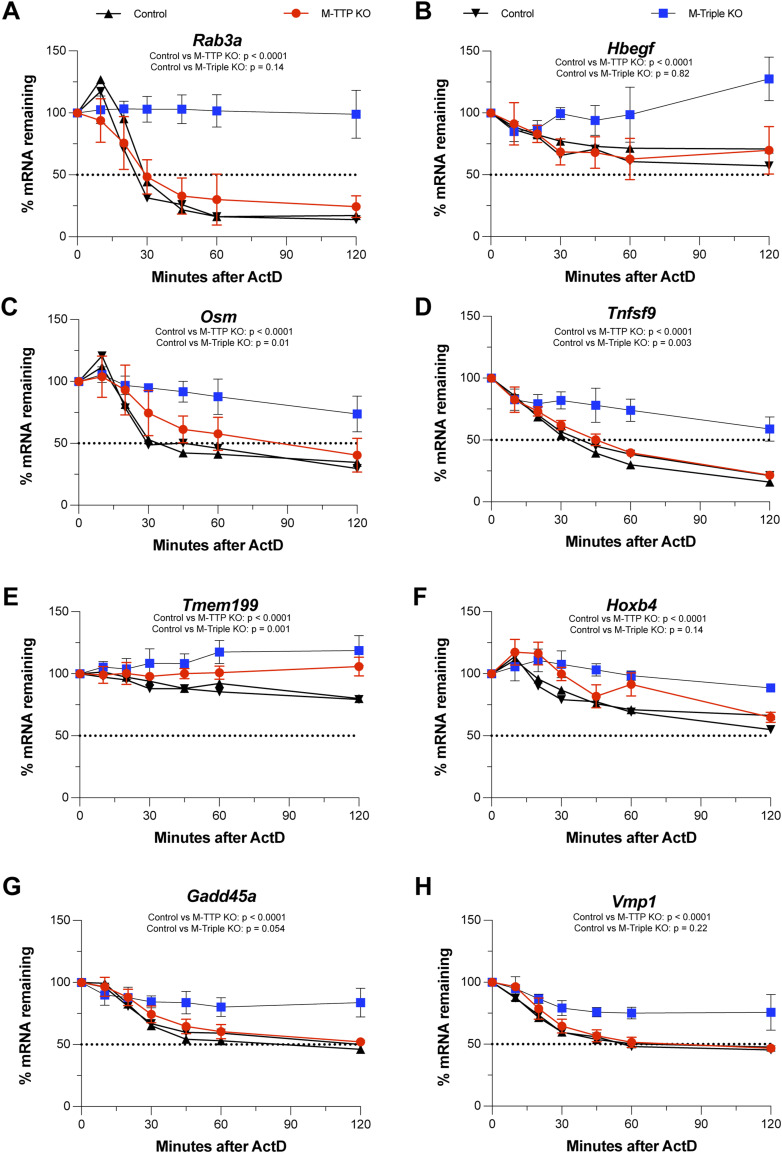
RNA-seq analysis of mRNA decay. BMDM from male 9-wk-old control, M-TTP KO, and M-Triple KO mice were cultured and treated with 1 μg/ml LPS for 1 h, followed by treatment with 5 μg/ml actinomycin D for various time points (cells were cultured from four animals in each group). Shown here are the top eight transcripts that were significantly stabilized at three consecutive time points in the M-triple KO macrophages, but not in the M-TTP KO macrophages compared with controls, as determined by unpaired two-tailed *t* tests. **(A, B, C, D, E, F, G, H)** Decay curves of (A) *Rab3a*, (B) *Hbegf*, (C) *Osm*, (D) *Tnfsf9*, (E) *Tmem199*, (F) *Hoxb4* (G) *Gadd45a*, and (H) *Vmp1* mRNAs in M-TTP KO and M-triple KO BMDM compared with control BMDM are shown as the percentages of mRNA remaining after 1 h of LPS treatment ± SD over 120 min after actinomycin D treatment. *P*-values are reported under the gene names as determined by Repeated Measures (RM) two-way ANOVA with the Geisser–Greenhouse correction and Šídák’s multiple comparison test.

Many transcripts appeared to be stabilized in M-triple KO macrophages but not in M-TTP KO macrophages (Fig 10). The top eight transcripts that were significantly delayed in the M-triple KO macrophages after LPS and ActD, but not in the M-TTP KO macrophages were as follows: *Rab3a* (Fig 10A), *Hbegf* (Fig 10B), *Osm* (Fig 10C), *Tnfsf9* (Fig 10D), *Tmem199* (Fig 10E), *Hoxb4* (Fig 10F), *Gadd45a* (Fig 10G), and *Vmp1* (Fig 10H). For example, there were no significant differences in the decay of *Rab3a* mRNA in M-TTP KO macrophages compared with controls (Fig 10A). In contrast, *Rab3a* mRNA was significantly stabilized in the M-triple KO macrophages, and its levels did not decrease appreciably during the full 120 min of the time course (Fig 10A).

Overall, some known TTP targets, such as *Tnf* mRNA, decayed slightly more slowly in M-TTP KO macrophages, but decayed much more slowly in M-triple KO macrophages after LPS and ActD treatment, suggesting at least an additive effect on TTP target mRNA stability. Some transcripts, such as *Rab3a* and *Hbegf* mRNAs, decayed significantly more slowly in M-triple KO macrophages, but not detectably in M-TTP KO macrophages, suggesting that *Zfp36l1* and *Zfp36l2* also play vital roles in regulating the stability of these transcripts in macrophages.

### Cytokine/chemokine levels in macrophage supernatants

**Figure S3.**
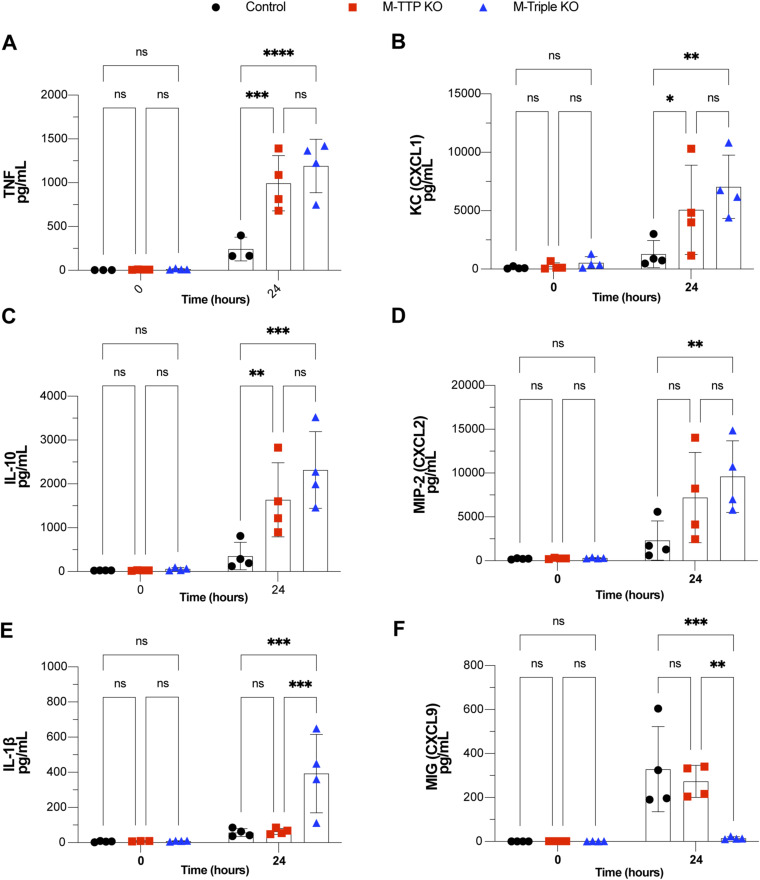
Cytokine analysis in BMDM supernatants. Analysis of cytokine/chemokine protein secretion into the medium of BMDM. BMDM from male 9-wk-old controls, M-TTP KO, and M-Triple KO mice were cultured and treated with 1 μg/ml LPS, and cell culture supernatants were collected after various time points of LPS treatment. **(A, B, C, D, E, F)** Levels of (A) TNF, (B) KC (CXCL1), (C) IL-10, (D) MIP-2 (CXCL2), (E) IL-1β, and (F) MIG (CXCL9) in the macrophage supernatant were determined by a plate-based immunoassay by EVE Technologies. *P < 0.05; **P < 0.01; ***P < 0.001; ****P < 0.0001; using two-way ANOVA with Šídák’s multiple comparison test.

Because many cytokine and chemokine mRNAs were apparently stabilized in the mutant macrophages compared with controls, we measured selected cytokines and chemokines in the culture medium of control and mutant macrophages. BMDM from control, M-TTP KO, and M-triple KO mice were treated with LPS, and then supernatants were used for the measurement of 31 cytokines and chemokines involved in inflammation. Levels of 3 of the 31 cytokines/chemokines were significantly increased in both the M-TTP KO and M-Triple KO macrophages when compared with controls after 24 h of LPS treatment: TNF (Fig S3A), CXCL1 (Fig S3B), and IL-10 (Fig S3C). MIP-2 (CXCL2) (Fig S3D) and IL-1β (Fig S3E) levels were both significantly increased in the M-triple KO macrophages after 24 h of LPS, but not in M-TTP KO macrophages (Fig S3D and E). MIG (CXCL9) levels in supernatants from M-triple KO macrophages were decreased when compared with control cells after 24 h of LPS treatment (Fig S3F), but were not significantly different from controls in the M-TTP KO cell supernatants.

## Discussion

### TTP family member transcripts in cultured WT BMDM before and after LPS stimulation

TTP (or ZFP36), ZFP36L1, and ZFP36L2 are paralogues that all emerged during early vertebrate evolution, and are products of different genes on different chromosomes (Taylor et al, 1991; Blackshear & Perera, 2014). In addition, as described above, knocking out the individual genes in mice led to very different phenotypes (Taylor et al, 1996; Stumpo et al, 2004, 2009). Nonetheless, they all have highly conserved tandem zinc finger and CNOT1 binding domains, and exhibit similar biochemical activities in RNA binding, deadenylation, and decay assays (Lai et al, 2000, 2003). This has raised questions as to whether the three proteins could be interchangeable or have redundant functions in certain circumstances. TTP is best known for its effects on pro-inflammatory cytokines and chemokines in myeloid cells (Carballo et al, 1998; Kratochvill et al, 2011; Qiu et al, 2012; Lee et al, 2017), but the potential functions and targets of ZFP36L1 and ZFP36L2 in myeloid cells are largely unknown.

In the experiments described here, basal levels of *Zfp36*, *Zfp36l1*, and *Zfp36l2* mRNAs were roughly equivalent in control mouse macrophages before LPS stimulation. However, *Zfp36* mRNA rapidly increased after 1 h of LPS stimulation and remained elevated even 24 h after the addition of LPS, whereas *Zfp36l1* and *Zfp36l2* mRNA levels slightly increased after LPS treatment and then rapidly decreased to below basal levels. These data, combined with area under the curve analyses, suggest that *Zfp36* mRNA is expressed to by far the greatest extent of the TTP family mRNAs after a pro-inflammatory stimulus in macrophages (Qiu et al, 2012). Previous experiments have shown that these changes in *Zfp36* mRNA levels are initially reflected in rapid and large changes in TTP protein accumulation, and it remains at high levels after LPS for many hours (Qiu et al, 2012, 2015; Patial et al, 2016a; Lai et al, 2018). Less is known about the behavior of the other two proteins under these conditions.

### Genome-wide effects of M-TTP KO and M-triple KO genotypes on transcript levels in unstimulated BMDM

In unstimulated BMDM from the M-TTP KO mice, TTP deficiency alone led to significant accumulation of only two transcripts by more than 1.3fold, of which only one (*Asb1*) contained one or more potential TTP binding sites (see Fig 8A and Table S1). A handful of transcripts were down-regulated under these conditions, presumed to be because of secondary or otherwise downstream events.

In contrast, measurement of steady-state transcript levels in the unstimulated M-triple KO macrophages revealed that 1,415 transcripts were significantly up-regulated by 1.3fold or more, including *Asb1*, and 1,223 transcripts were significantly down-regulated by 1.3fold or more, when compared with control macrophages (see Fig 8C and Table S3). There was significant enrichment of potential TTP binding site sequences in the up-regulated transcripts compared with the down-regulated transcripts, and many of the up-regulated transcripts were previously known or suspected targets of TTP (Brooks & Blackshear, 2013; Patial et al, 2016b). We presume that there are many other direct targets of TTP and its paralogues in this collection, and that the rest of the up-regulated, and probably all of the down-regulated, transcripts are the results of secondary or otherwise downstream events.

Although much previous work has demonstrated that the presence of at least one TTP binding site is necessary for TTP to bind to and destabilize its target transcripts (Carballo et al, 1998; Lai et al, 1999; Lai et al, 2000; Lai and Blackshear, 2001; Lai et al, 2003; Brewer et al, 2004; Lai et al, 2005; Qiu et al, 2015; Lai et al, 2018), many apparently unaffected or down-regulated transcripts in the present experiments contain potential TTP family member binding sites within their 3′-UTRs, but did not appear to be stabilized. This phenomenon has been observed in experiments of this type in many different cell types, and there is no simple explanation (Patial et al, 2016b). One possibility is that the potential binding sites could be occupied by other AU-rich element-binding proteins or even other RNAs (Sobolewski et al, 2022). Another possibility is that there could be an RNA secondary structure involving the potential binding site, which has been shown to prevent binding by TTP family proteins (Hudson et al, 2004). TTP and its family members also may occupy specific locations within the cytosol that may not expose them to the relevant mRNAs (Phillips et al, 2002). There may be multiple mechanisms at work, but the general consensus from many experiments is that the number of transcripts with potential binding sites is much greater than those demonstrated to decay in response to TTP-like proteins.

What is the mechanism of this remarkable difference in transcript level changes between the unstimulated cells from the M-TTP KO and M-triple KO mice? The most likely mechanism is that the three TTP paralogues, present at similar levels, act on the same biochemical pathways in the WT cells. In this proposed mechanism, the proteins would bind to the same AU-rich motifs in the same transcripts, and promote their deadenylation and decay, in a synergistic fashion. One corollary of this idea is that the three proteins are behaving essentially identically as “TTP equivalents.” This will be difficult to prove, but it is supported by the similar effects exhibited by the proteins in cell transfection mRNA decay assays, and cell-free assays of RNA binding and promotion of deadenylation (Lai et al, 2000, 2003). On the other hand, there have been several reports of, for example, TTP-specific activities based on primary amino acid sequences that are unique to TTP (Lykke-Andersen & Wagner, 2005; Bulbrook et al, 2018). Ongoing studies are underway to test the “equivalence” concept, including expressing comparable levels of the three proteins in cells, and determining whether the induced changes in gene expression are identical or different. We are also extending the present studies to investigate the effects of knocking out two of the three family member genes on gene expression patterns under the same conditions.

It should be noted that the macrophages used in our experiments were incubated in low-serum medium for 16–18 h before harvesting, so some changes in actual target transcripts, and at least some of the presumed downstream effects, may be in part because of autocrine/paracrine effects of secreted cytokines such as TNF, whose transcript is a well-known target of TTP in these cells.

### Phenotypes of M-triple KO mice compared with control mice, and single myeloid-specific KO mice, under normal vivarium conditions

As described previously for M-TTP KO (Kratochvill et al, 2011; Qiu et al, 2012) and M-ZFP36L1 KO (Hyatt et al, 2014) mice, the single myeloid-specific KO mice for each of the three genes tested in this study did not exhibit any weight loss, arthritis, premature death or any other external phenotypes in the first several months of age. In marked contrast, the M-triple KO mice had a severe, spontaneous inflammatory phenotype, with marked peripheral arthritis, failure of weight gain, and early death, with median survivals of only about 8 wk for both sexes. This was characterized by increased levels of many pro-inflammatory cytokines in the serum, and histological evidence of widespread, severe soft tissue infiltration by inflammatory cells, bone destruction and osteopenia, splenomegaly, and many other pathological changes. Overall, the arthritis in both the whole-body *Zfp36*-KO mice and the M-triple KO mice is similar to the pathology observed in human rheumatoid arthritis (Komatsu & Takayanagi, 2022) and in mouse models of rheumatoid arthritis, such as collagen antibody-induced arthritis (Caplazi et al, 2015).

As detailed above in the Results section, several cytokines and chemokines were elevated in the the serum of the M-triple KO mice, and many of these were also found in the medium of their cultured BMDM. Many of these are undoubtedly involved in the pathogenesis of the severe M-triple KO syndrome, and their involvement in inflammatory diseases and disease models has been documented in many previous studies (Deshmane et al, 2009; Lee et al, 2017; Karin & Razon, 2018; Steinkamp et al, 2018; Tokunaga et al, 2018; House et al, 2020; Yao et al, 2021). On the other hand, some well-known pro-inflammatory cytokines, such as G-CSF and IL-6, were increased in the serum of M-triple KO mice, but not in serum from M-TTP KO mice. These have also been implicated in previous models of inflammatory disease, and in myeloid lineage specification (Lawlor et al, 2004; Roberts, 2005; Kaplan et al, 2011; Theyab et al, 2021).

It seems probable that the pathogenetic mechanism for this phenotype is a consequence of the gene expression changes discussed above in unstimulated macrophages. Specifically, even under normal vivarium conditions, there is likely to be increased stabilization of many transcripts encoding pro-inflammatory cytokines in myeloid cells; this is reflected in the increases we saw in serum cytokine and chemokine levels, and increased levels of these proteins in culture media from the KO cells. TNF is a good example of how the gene expression changes seen in the unstimulated, triple KO macrophages are reflected in mouse phenotype. *Tnf* mRNA levels were not elevated in the unstimulated macrophages from the M-TTP KO mice, but were significantly increased by 3.5fold in the M-triple KO macrophages. This was reflected in increases in TNF protein levels in serum from the M-triple KO mice, even without innate immune stimulation. The severe phenotype observed in the M-triple KO mice could therefore be in part because of a combination of direct pathogenic effects of the elevated TNF, and autocrine/paracrine effects of TNF to promote the secretion of many other pro-inflammatory cytokines, including itself (Grivennikov et al, 2005; Kruglov et al, 2008; Moudgil & Choubey, 2011; Steinkamp et al, 2018; Yao et al, 2021). Similar severe phenotypes have been observed in mice with direct transgenic overexpression of TNF (Keffer et al, 1991; Probert et al, 1993), or mice in which an AU-rich instability region of the *Tnf* mRNA have been removed, resulting in systemic TNF overexpression (Kontoyiannis et al, 1999). It will be of great interest to see whether the M-triple KO phenotype can be modified by interfering with TNF activity, as has been done in the case of the conventional TTP KO mice (Taylor et al, 1996; Carballo & Blackshear, 2001), and other pro-inflammatory pathways that have been shown to have an ameliorating effect on the whole-body TTP-deficiency syndrome (Molle et al, 2013).

Examples of how this might work in the present study come from several groups of genes whose expression was altered in the M-triple KO macrophages in the absence of stimulation. For example, levels of 15 chemokine transcripts were significantly increased by more than 1.3fold in the unstimulated M-triple KO macrophages, with one of them, *Ccl12* mRNA, increased by 238fold (adjusted *P*-value 6.02 × 10^−11^). This transcript contained a single potential TTP family binding site, but two other highly up-regulated chemokine transcripts, *Ccl7* (46fold increase) and *Ccl8* (37fold increase) mRNAs, did not contain obvious binding sites, and may be examples of autocrine/paracrine secondary stimulation. Similarly, five transcripts from the *Cxcl* family of chemokines were increased, but only two of them, *Cxcl1* and *Cxcl2* mRNAs, are well-known TTP targets (Jalonen et al, 2005; Datta et al, 2008; Qiu et al, 2015), whereas the other three do not contain obvious TTP family binding sites. A final example is interferon β1 (*Ifnb1*) mRNA, whose levels were increased by about 10.5fold. This transcript has a single potential TTP binding site and was the only interferon transcript up-regulated in this collection. Perhaps in response, there were 22 interferon-activated or -induced transcripts among the up-regulated mRNAs, of which only *Ifit1* mRNA (increased 15fold) contained an obvious TTP binding site.

Taken together, the data suggest that the severe phenotype of the M-triple KO mice was because of a combination of primary increased synthesis and secretion of certain pro-inflammatory proteins whose transcripts are direct targets of TTP family proteins, such as *Tnf* mRNA, and secondary effects of those primary pro-inflammatory proteins on the myeloid cells themselves and other cells and tissues that they influence.

### Transcript stability changes in LPS-stimulated macrophages from M-TTP KO mice compared with cells from M-triple KO mice

We attempted to measure mRNA decay rates in WT, M-TTP KO, and M-triple KO macrophages to identify likely primary targets for TTP and its family members in this system, and to look for additivity or synergy between the family members. As described above, both *Zfp36l1* and *Zfp36l2* mRNA levels decreased rapidly to below baseline levels after LPS stimulation of WT macrophages, and it seemed possible that the high remaining levels of TTP in this situation would result in transcripts from the M-triple KO cells decaying at the same rate as those from the M-TTP KO cells.

However, this was not the case. Under the stringent criteria we used to measure changes in mRNA decay, transcripts could be divided into several groups. The largest group included transcripts in which no stability differences could be measured; most of these transcripts decayed too slowly in the control cells to be included in the analysis, and a smaller subset decayed fast enough to be analyzed but exhibited no differences among the three genotypes. However, for those transcripts in which significant differences in decay rates could be measured, three major groups could be delineated. In the first group, stability of transcripts from M-TTP KO cells did not differ from transcript stability in control cells, but stability was significantly increased when the M-triple KO cells were compared with control cells. The most striking examples of transcripts in this group are illustrated in Fig 10. For example, *Rab3a* mRNA decayed at the same rate as control in the M-TTP KO cells, but was completely stabilized in the M-triple KO cells. This transcript contains four potential TTP family member binding sites, some of them overlapping, and its encoded protein is involved in GTP-dependent protein binding, especially in neuronal processes (Geppert et al, 1997; Sheehan et al, 2016). Its roles in myeloid cells are unclear, but previous studies have suggested a role in plasma membrane repair and lysozyme exocytosis in response to bacterial toxins (Abu-Amer et al, 1999; Vieira, 2018). In the present situation, it is the clearest example of a transcript whose normal instability seems to be unaffected by TTP deficiency, but is completely stabilized in the absence of all three family members. As such, it may be a good marker transcript to use in addressing future questions about specificity among the family members, for example, is it a specific target for one of the three proteins, or is its normal processsing dependent on the presence of normal levels of all three proteins, acting in concert and perhaps synergistically?

In a relatively small group of transcripts, the mRNA decay rates were slowed to approximately the same extent in both the M-TTP KO and M-Triple KO cells. Good examples of members of this group are *Cxcl2* and *Ccl2* mRNAs, as shown in Fig 9. Both mRNAs have been described previously as TTP targets (Jalonen et al, 2005; Sauer et al, 2006; Qiu et al, 2015), and both encode important chemokines involved in leukocyte chemotaxis and pathogenesis of inflammatory diseases (Sauer et al, 2006; Deshmane et al, 2009; De Filippo et al, 2013).

A final group consists of transcripts in which significant stabilization occurred in the setting of TTP deficiency alone, but was increased further in the M-triple KO cells. This group contains some of the best known targets of TTP in these cells (Taylor et al, 1996; Carballo et al, 1998, 2000; Lai et al, 2006; Datta et al, 2008), including *Tnf*, *Ier3*, *Cxcl1*, and *Csf2* mRNAs (see Fig 9). In these cases, the effects of knocking out all three family members appeared closer to additive.

Given the behavior of *Zfp36l1* and *Zfp36l2* transcripts in the control cells after LPS stimulation, that is, decreases below starting levels within a short period after LPS, it is difficult to explain their ability to contribute to the decay rates of the transcripts described above in the control cells. One possible explanation has to do with the fact that their levels are approximately the same as *Zfp36* mRNA levels in the pre-stimulated, steady-state condition, possibly resulting in similar levels of all three encoded proteins that could persist for the relatively short duration of the ActD experiment. These protein levels could actually increase in response to the rapid, transient peaks in mRNA levels seen in the short term after LPS, and then persist for the duration of the decay experiment, despite the apparent suppression of their transcripts. Other explanations are possible, including changes in phosphorylation status, interactions with cytoplasmic binding proteins, and others. It seems clear, however, that the three encoded proteins, acting together, are often more potent in promoting mRNA decay in these experimental circumstances than TTP alone.

### Physiological importance of the three expressed TTP family members in myeloid cells

An advantage of the severe, universal, early onset phenotype seen in the M-triple KO mice is that it provides an assay for the contributions of the different family members to the phenotype. Although the numbers of mice were small in each group, we found that two normal alleles of any of the three genes, in the setting of myeloid cell deficiency of the other two, could completely prevent the early death, growth inhibition, and peripheral arthritis seen in the M-triple KO mice. Even one normal allele of *Zfp36* or *Zfp36l2*, in the absence of all others, could prevent the development of the syndrome, whereas one normal allele of *Zfp36l1* in the absence of the others led to a less severe version of the M-triple KO phenotype. This is additional evidence that the protein activities of the three paralogues are to some extent overlapping and redundant.

In contrast to the situation in mice living under normal vivarium conditions, the M-TTP KO mice were extremely susceptible to small doses of LPS, and died of a sepsis-like syndrome that their WT counterparts survived without difficulty (Kratochvill et al, 2011; Qiu et al, 2012). It will be of great interest to see how the apparently phenotypically normal mice that express only one of the three genes will respond to this kind of inflammatory stimulus. It will also be interesting to correlate those results with the effects of the double KOs on transcript expression and stability in the presence and absence of LPS. These types of experiments are currently under way.

LysM-Cre has been shown to provide conditional KOs in several different types of myeloid cells (Clausen et al, 1999), and it will be of great interest to try to dissect the effects of deficiencies of the different family members in specific cell types, using cell type-specific Cres. Another approach to the question of cell specificity might be to sort the various cell types present in the bone marrow and other tissues from the M-TTP KO and M-triple KO mice, and examine their gene expression patterns and transcript stability with or without an inflammatory stimulus.

Although the phenotype of the M-triple KO mice was very severe and affected many organ systems, it was almost as striking to note abnormalities that might be expected that did not occur. For example, most models of TNF excess, either by transgenic overexpression or by deletion of the AU-rich instability element, are associated with a severe form of inflammatory bowel disease (Kontoyiannis et al, 1999). We have never seen this in the conventional TTP KO mice (Taylor et al, 1996), nor was it present in the M-triple KO mice described here, despite evidence of TNF overexpression from macrophages and presumably other myeloid cells. Similarly, a severe form of ocular inflammation leading to blindness was seen in the T-cell conditional triple KO mice described recently by Cook et al (2022), whereas we did not observe eye abnormalities in the M-triple KO mice. In their study, Cook et al (2022) found that mice with simultaneous deletion of *Zfp36*, *Zfp36l1*, and *Zfp36l2* in T cells under the control of the *Cd4*-Cre developed a lethal inflammatory syndrome with multi-organ involvement and overproduction of inflammatory cytokines (Cook et al, 2022).

Previous work has shown that mice with double KO of *Zfp36l1* and *Zfp36l2* in T cells during thymopoiesis develop T cell acute lymphoblastic leukemia because of changes in Notch-1 signaling (Hodson et al, 2010). However, mice with single KO of *Zfp36l1* or *Zfp36l2* individually in T cells during thymic development do not develop T cell acute lymphoblastic leukemia, suggesting that *Zfp36l1* and *Zfp36l2* may have redundant functions in T cells (Hodson et al, 2010). Mice with deletion of either *Zfp36l1* or *Zfp36l2* individually in muscle cells using Pax-7 Cre did not display any growth defects or phenotype, yet mice with deficiency of *Zfp36l1* and *Zfp36l2* simultaneously in Pax7-expressing satellite cells had reduced body weight, reduced skeletal muscle mass, and reduced capacity to regenerate muscle after muscle injury (Bye et al, 2018).

In summary, the complete absence of all three expressed TTP family members from myeloid cells in mice led to a spontaneous, severe inflammation syndrome that affected many organ systems. This could be prevented by normal expression of any of the three family member genes, and, in two instances, by only a single allele of those genes. This shows a remarkable degree of redundancy among the three expressed family members, and may help explain certain experiments of nature, like the ability of birds to survive despite their apparent lack of TTP expression (Lai et al, 2013). Many questions remain unanswered, but among the most important is the question of specificity and cross-reactivity among the different family members, both in cell-free experiments with purified components of the mRNA deanylation apparatus, and in intact cells.

## Materials and Methods

### Mice

Mice with *lox*P sites flanking the second exon of each respective gene (*Zfp36*^*flox/flox*^, *Zfp36l1*^*flox/flox*^, or *Zfp36l2*^*flox/flox*^) were generated by gene targeting in C57Bl/6 embryonic stem cells by Xenogen Biosciences, as previously described (Qiu et al, 2012; Hyatt et al, 2014; Dumdie et al, 2018). Expression of Cre recombinase under the control of the murine M lysozyme promoter (LysM-Cre) was used to effect deletion specifically in cells of the myeloid lineage (monocytes, macrophages, and granulocytes). Homozygous LysM-Cre (B6.129P2-*Lyz2*^*tm1(cre)Ifo*^) mice on a C57Bl/6 background were purchased from the Jackson Laboratory (JAX stock #018956) (Clausen et al, 1999; Takeda et al, 1999). Individual KO mice for each TTP family member were generated by crossing either *Zfp36*^*flox/flox*^, *Zfp36l1*^*flox/flox*^, or *Zfp36l2*^*flox/flox*^ mice with LysM-Cre mice. For experiments using *Zfp36*^flox/flox^; *LysM-Cre*^+/−^ mice, *Zfp36*^flox/flox^; *LysM-Cre*^−/−^ littermates were used as controls.

A mouse line in which all three TTP family members were floxed, referred to as “triple floxed” mice, was generated by inter-crossing and breeding *Zfp36*^*flox/flox*^, *Zfp36l1*^*flox/flox*^, and *Zfp36l2*^*flox/flox*^ mice to homozygosity for the floxed alleles of *Zfp36*, *Zfp36l1*, and *Zfp36l2*. Next, myeloid-specific triple (M-triple) KO mice were achieved by crossing the triple-floxed (*Zfp36*^*flox/flox*^; *Zfp36l1*^*flox/flox*^; *Zfp36l2*^*flox/flox*^) mice with LysM-Cre mice. In this breeding scheme, various myeloid-specific (M-double) KO combinations were also generated. The deletion of *Zfp36*, *Zfp36l1*, *Zfp36l2* in macrophages was confirmed (data not shown) and the efficiency of the LysM-Cre in myeloid cells has been previously reported (Clausen et al, 1999; Takeda et al, 1999; Qiu et al, 2012). For experiments using *Zfp36*^flox/flox^; *Zfp36l1*^flox/flox^; *Zfp36l2*^flox/flox^; *LysM-Cre*^+/−^ mice, *Zfp36*^flox/flox^; *Zfp36l1*^flox/flox^; *Zfp36l2*^flox/flox^; *LysM-Cre*^−/−^ littermates were used as controls.

### Tissue processing

Mice were euthanized at 8–10-wk-old, gross examinations of all organs were performed, and the following tissues were harvested: adrenal gland, brain with olfactory nerve, cervix, esophagus, eyes with optic nerve, femur, gallbladder, Harderian glands, heart/aorta, small and large intestines (duodenum, jejunum, ileum, cecum, colon, rectum), kidney, liver, lungs, lymph nodes, mammary gland, skeletal muscle, sciatic nerves, nose, nasal cavity, ovary, pancreas, pituitary gland, prostate gland, salivary gland, seminal vesicle, haired skin, spinal cord, spleen, stomach (glandular and nonglandular), testes, thymus, thyroid gland, tongue, trachea, urinary bladder, uterus, and vagina. Internal organs were fixed in 10% neutral-buffered formalin and used for paraffin embedding, sectioning, and hematoxylin and eosin staining. Tissue samples containing bones, such as paws, were decalcified with Immunocal (StatLab) for greater than 8–36 h. Microscope images were captured using a Hamamatsu C13220 Microscope with a 20X objective with a 40X lens and 0.75 aperture. The magnification of each image is specified in the respective figure legend.

### Peripheral blood analysis

For hematological analysis, peripheral blood was collected by cardiac puncture, collected into K3-EDTA-containing Microvette blood collection tubes (Sarstedt), and analyzed using a Procyte Hematology Analyzer (IDEXX).

### Measurement of cytokine levels in serum

Peripheral blood was collected by cardiac puncture into a Micro Z-Gel serum separation tube (Sarstedt), followed by centrifugation for 5 min at 10,000*g* at RT, after which the supernatant was collected. Cytokine and chemokine analyses were performed using a Mouse Cytokine/Chemokine 31-Plex Array (Eve Technologies).

### MicroCT

Front and hind paws were fixed in 10% neutral-buffered formalin for 72 h, followed by storage in 70% ethanol. The paws were removed from alcohol storage and mounted within a sealed plastic tube sample holder (Corning) with internal physical anchoring by foam to prevent movement during scanning. The samples were scanned using a SKYSCAN 1272 (Bruker microCT) at a nominal resolution of 6.5 microns, using a 0.25-mm-thick aluminum filter, and an applied x-ray voltage of 65 kV and 153 μA. Camera pixel binning of 2 × 2 was applied. The scan orbit was 360° with a rotation step of 0.32°. Reconstruction was carried out with a modified Feldkamp algorithm using the SkyScan NRecon software accelerated by a graphics processing unit. Appropriate ring artefact reduction and 30% beam hardening correction were applied. A SkyScan CT-Analyzer software suite was used for 3D model reconstructions and bone morphometric analyses. Reconstructed data were volume rendered in CTVoxx (Bruker microCT) and a custom transfer function was created to completely reduce the tissue opacity from the window/level of view. In addition, an RGB color scale was applied to the data to better translate the surface of the bone. All samples were consistently handled such they all received the same transfer function, RGB, and lighting settings.

### Flow cytometry analysis

8–10-wk-old mice were euthanized by CO_2_ inhalation, and femurs were dissected. Bone marrow cells were isolated by flushing dissected femurs with 5 ml PBS containing 2% FBS, using a 25-gauge needle. The cell suspension was filtered over a 100-μM nylon mesh strainer (Thermo Fisher Scientific), followed by centrifugation for 5 min at 500*g* at 4°C. Cells were resuspended as a single cell suspension, and 1 × 10^5^ cells were stained with 25 μl of Brilliant Stain Buffer (BD Biosciences) and the following antibodies for 30 min at 4°C in the dark: BV421–anti-CD45, AF488–anti-CD3, PE–anti-CD4, AF647–anti-CD8, PE-Cy7–anti-CD19, AF700–anti-CD11b, PerCP-Cy5.5–anti-CD11c, BV480–anti-MHC-II, APC-Cy7–anti-Ly-6G, PE-CF594–anti-Ly-6C (BD Biosciences). Red blood cells were lysed with FACSLyse (BD Biosciences), followed by centrifugation for 5 min at 500*g*, and resuspension in staining buffer. Data were acquired on an LSRII Flow Cytometer (BD Biosciences) and analyzed with FlowJo software (FlowJo BD).

For the analysis of hematopoietic stem and progenitor cells, 1 × 10^7^ bone marrow cells were treated with 1 ml ACK lysis buffer (0.1 M NH_4_CH_3_CO_2_, 10 mM KHCO_3_, 0.1 mM EDTA, produced by NIEHS Media and Glassware Units) at RT for 30 s to deplete red blood cells, then quenched with staining medium (PBS supplemented with 2% FBS and 2 mM EDTA). Cells were then centrifuged at 300*g* for 5 min, resuspended in the staining medium, and stained on ice for 30 min with the following antibodies: CD117 (c-Kit) APC (105808; BioLegend), Sca-1 APC-Cy7 (108126; BioLegend), CD3 Biotin (100244; BioLegend), Ly-6G/Ly-6C (Gr-1) Biotin (108404; BioLegend), B220 Biotin (103204; BioLegend), TER-119 Biotin (116204; BioLegend), CD11b Biotin (101204; BioLegend), CD150 (SLAM) PE-Cy7 (115914; BioLegend), CD41 FITC (133904; BioLegend), CD105 (Endoglin) PerCP/Cyanine5.5 (120416; BioLegend), CD16/32 (FcγRII/III) eFluor 450 (48-0161-82; eBioscience). After the primary staining, cells were then washed once, incubated with Streptavidin APC (SA1005; Invitrogen) on ice for 15 min to reveal biotin-conjugated lineage antibodies, and then resuspended at 5 × 10^6^ cells/ml for flow cytometry. Data were collected using an LSRFortessa instrument (BD Biosciences) and analyzed using BD FACSDiva and FlowJo (BD Life Sciences).

The following surface marker combinations were used to identify adult HSPC subtypes (Nakorn et al, 2003; Pronk et al, 2007; Weksberg et al, 2008; Cabezas-Wallscheid et al, 2014): HSC (Lin−, Sca1+, c-Kit+, CD150+); multipotent progenitor (Lin−, Sca1+, c-Kit+, CD150−); Mye Pro(Lin−, Sca1−, c-Kit+); MKP (Lin−, Sca1−, c-Kit+, CD150+, CD41^+^); PreMegE (Lin−, Sca1−, c-Kit+, CD41−, CD16/32−, CD150+, Endoglin−); Pre CFU-E (Lin−, Sca1−, c-Kit+, CD41−, CD16/32−, CD150+, Endoglin+); CFU-E and Pro Ery (Lin−, Sca1−, c-Kit+, CD41−, CD16/32−, CD150−, Endoglin+); PreGM (Lin−, Sca1−, c-Kit+, CD41−, CD16/32−, CD150−, Endoglin−); granulocyte–macrophage progenitor (Lin−, Sca1−, c-Kit+, CD41−, CD16/32+).

### Culture and treatment of BMDM

8–9-wk-old mice were euthanized by CO_2_ inhalation, and both femurs were isolated aseptically. In a tissue culture hood, both femurs from each animal were flushed with RPMI 1640 Medium (Gibco; Thermo Fisher Scientific), using a 25-gauge needle over a 100-μM nylon mesh strainer (Thermo Fisher Scientific), followed by centrifugation for 5 min at 500*g* at RT. The pelleted cells from each animal were resuspended and plated into five, 10 cm TC-treated culture dishes (Corning) and cultured in RPMI 1640 Medium (Gibco; Thermo Fisher Scientific) supplemented with 30% L929-conditioned medium, 10% ES Cell-screened FBS (HyClone; Cytiva), 15 mM HEPES (Sigma-Aldrich), 2 mM glutamine, and 100 U; 100 μg/ml penicillin/streptomycin (Gibco; Thermo Fisher Scientific). On day 4 after plating the cells, the floating cells remaining in suspension were collected and plated onto new plates for experiments. Supplemental medium was added every other day until the cells reached ∼80% confluency around days 7–10 (Qiu et al, 2012; Lai et al, 2019b, 2019c).

Once reaching confluency, BMDM were then incubated for 16–18 h in a serum starvation medium (RPMI supplemented with 1% FBS, 2 mM glutamine, 100 U penicillin/100 μg/ml streptomycin), followed by treatment with 1 μg/ml LPS (L6529; Sigma-Aldrich) and/or ActD (A4262; Sigma-Aldrich) for the designated time points (Lai et al, 2019a). For mRNA decay experiments, four mice of each genotype were used, and three plates of cells from each mouse were pooled for each time point. For mRNA accumulation experiments, four plates of cells were pooled and used as controls (untreated), and three plates of cells were pooled and used for each time point of LPS treatment (1 μg/ml).

### RNA extraction

Total cellular RNA was isolated from cultured bone marrow macrophages using the illustra RNAspin RNA isolation kit, according to the manufacturer’s instructions (GE Healthcare). The RNA content and purity were determined by measuring absorbance at 260/280 nm on NanoDrop One (Thermo Fisher Scientific) and the quality of RNA was determined using TapeStation System (Agilent).

### NanoString RNA analysis

Total cellular RNA from BMDM used in the RNA accumulation experiments was analyzed using the NanoString nCounter method (Fortina & Surrey, 2008) for *Zfp36*, *Zfp36l1*, and *Zfp36l2* mRNAs. The observed counts were normalized by a series of internal spike-in controls, and a set of housekeeping controls that had been previously validated as stable during LPS treatment.

### Library preparation and RNA sequencing

Library preparation was performed using the TruSeq RNA Library Prep Kit (Illumina Inc.) at the NIEHS Epigenomics Core Laboratory. RNA deep-sequencing was performed using 75-bp paired-end reads on the NovaSeq6000 platform (Illumina Inc.) at the NIEHS Epigenomics Core Laboratory.

### RNA-seq data analysis

RNA-seq data analysis was performed by beginning with fastq sequence files of 75-base pair-ended reads and subjecting them to read quality and length trimming using bcl2fastq2. Read pairs were filtered to remove those in which either mates’ mean quality score fell below 20. 50 million reads per sample were aligned to the mm10 transcriptome using STAR 2.7.0f. Counts per gene were determined using featureCounts 1.5.1. Differentially expressed transcripts were identified by DESeq2 using filtered thresholds of FDR < 0.05 and log_2_ fold change ≥ 0.3785 (or a raw fold change of 1.3), with the additional requirement of a mean control FPKM > 0.1 and an adjusted *P*-value ≤ 0.05.

### Analysis of mRNA decay from RNA-seq data

mRNA decay curve analysis was performed by first removing non-mRNAs, the knocked out genes (*Zfp36*, *Zfp36l1*, and *Zfp36l2*), the artefactually up-regulated gene *Plekhg2*, mRNAs whose mean levels in the control cells were < 0.1 FPKM, and mRNAs that did not decrease below 85% mRNA remaining in control BMDM after 120 min of ActD. The data were then converted to the percentage of the original mRNA remaining after the 1 h LPS time point. To narrow down the list of transcripts to analyze the decay curves, an initial screen was performed using two-tailed *t* tests with Bonferroni correction between the control and M-TTP KO cells or the control and M-triple KO cells at three consecutive time points, with a cutoff of *P* < 0.008. After this initial list of candidates was identified, two-way ANOVA with Geisser–Greenhouse correction and Šídák’s multiple comparison test was performed with a cutoff of an ANOVA *P*-value < 0.05, used as a more stringent statistical test to identify genotype differences between the full decay curves. The apparently stabilized transcripts were then ranked by the biggest difference in the average percent mRNA remaining at the 60- and 120-min time points between the control and M-TTP KO samples, or the control and M-triple KO samples. One thing to note is that addition of ActD causes a large number of transcripts to decay rapidly, resulting in a decline in the total RNA isolated at later time points. Standard normalization methods used in RNA-seq assume that libraries are prepared from comparable pools of RNA. Therefore, levels of non-decaying or slowly decaying transcripts will artefactually appear to increase (Lai et al, 2019a). However, in the experiments described above, each pair of WT and KO cells was treated in the same experiment, and any drift in the baselines should be comparable between the genotypes throughout the experiment.

### Identification of TTP binding sites

A custom TTP binding site search application (available upon request) was used to scan the 3′-UTR of potential target transcripts for the presence of possible high-affinity TTP binding sites: UAUUUAU (7-mer), UAUUUUAU (8-mer), UUAUUUAUU (9-mer), and UUAUUUUAUU (10-mer), as previously described (Lai et al, 2013; Patial et al, 2016b).

### IPA

The differentially expressed transcripts were subjected to IPA to investigate the biological networks and pathways that were enriched under these conditions (QIAGEN).

### Statistical analysis

Results are expressed as means ± SD unless otherwise specified. The statistical significance was assessed as indicated using GraphPad Prism 9.0. The following tests were used: unpaired, two-tailed *t* tests with Welch’s correction, one-way ANOVA, and two-way ANOVA test with Šidák’s multiple comparison test. A *P*-value less than 0.05 was considered significant.

### Study approval

All animal breeding and other procedures were approved by the Institutional Animal Care and Use Committee of the National Institute of Environmental Health Sciences. Animals were maintained in a specific pathogen-free facility with ad libitum access to food and water.

## Data Availability

The data discussed in this publication are accessible through NCBI GEO Series accession number GSE229924.

## Supplementary Material

Reviewer comments
